# ALA reverses ABA-induced stomatal closure by modulating PP2AC and SnRK2.6 activity in apple leaves

**DOI:** 10.1093/hr/uhad067

**Published:** 2023-04-10

**Authors:** Zheng Chen, Yuyan An, Liangju Wang

**Affiliations:** College of Horticulture, Nanjing Agricultural University, Nanjing 210095, China; College of Life Sciences, Shaanxi Normal University, Xi’an 710119, China; College of Horticulture, Nanjing Agricultural University, Nanjing 210095, China

## Abstract

5-Aminolevulinic acid (ALA), known as a new natural plant growth regulator, can reverse abscisic acid (ABA)-induced stomatal closure. The protein phosphatase 2A (PP2A) played an important role in regulation of stomatal movement by ALA and ABA; however, the underlying molecular mechanisms remain unclear. Here, we report that ALA promotes MdPP2A activity and gene expression in the leaf epidermis of apple (*Malus × domestica* Borkh.), and expression of the catalytic subunit *MdPP2AC* was most significantly correlated with stomatal aperture. Western blotting showed that ALA enhanced MdPP2AC protein abundance and phosphorylation. Y2H (yeast two hybrid), FLC (firefly luciferase complementation imaging) and BiFC (Bimolecular fluorescence complementation) assays showed that MdPP2AC interacted with several other MdPP2A subunits as well as MdSnRK2.6 (Sucrose non-fermenting 1-related protein kinase 2.6), and the latter interaction was further verified by pull-down and MST (microscale thermophoresis) assays. ALA downregulated ABA-induced *MdSnRK2.6* gene expression, kinase activity, and protein phosphorylation. In transiently transgenic apple leaves, OE-*MdPP2AC* promoted stomatal aperture by reducing Ca^2+^ and H_2_O_2_ levels but increasing flavonol levels in guard cells. Conversely, OE-*MdSnRK2.6* induced stomatal closure by increasing Ca^2+^ and H_2_O_2_ but reducing flavonols. Partial silencing of these genes had opposite effects on Ca^2+^, H_2_O_2_, flavonols, and stomatal movement. Application of exogenous ALA stimulated PP2A activity, which promoted SnRK2.6 dephosphorylation and lower kinase activity in wild-type and transgenic apple leaves. We therefore propose that PP2AC, which dephosphorylates SnRK2.6 and represses its enzyme activity, mediates ALA signaling to inhibit ABA-induced stomatal closure in apple leaves.

## Introduction

5-Aminolevulinic acid (ALA), a natural δ-amino acid, is not involved in protein biosynthesis but instead acts as an essential biosynthetic precursor of all tetrapyrrole compounds such as chlorophylls and hemes [1], which are closely associated with plant photosynthesis [2] and respiration [3]. In the past two decades, ALA has been proposed as a new natural plant growth regulator [4] that can promote plant growth and crop yield [5], as well as improve stress resistance [2, 6]. Because it is environmentally friendly with multiple biological regulatory functions, ALA has been suggested to have wide applications in agriculture and horticulture [7–12]. In apple fruits, ALA effectively promotes the accumulation of anthocyanins [13–16] and flavonols [17].

Among its multiple functions, the basic role of ALA is the improvement of photosynthesis, as it can promote and regulate the biosynthesis of chlorophylls [4, 5, 10]. Another important mechanism by which ALA can improve photosynthesis is by increasing stomatal conductance, which enhances CO_2_ entry into mesophyll cells. This effect was first observed in melon leaves when plants were stressed under low light and chilling conditions [2]. Since then, many studies have confirmed that ALA inhibits dark- or abscisic acid (ABA)-induced stomatal closure by reducing hydrogen peroxide (H_2_O_2_) and calcium (Ca^2+^) concentrations while increasing flavonol levels in guard cells [18–21]. ALA can therefore antagonize stress-induced ABA to maintain stomatal opening, attenuating stomatal limitations on CO_2_ uptake. This has particular significance for the maintenance of photosynthesis under stress [11], but the specific mechanisms by which ALA regulates stomatal movement are largely unknown.

Stomatal movement is affected by many factors. Previous studies by our research group have shown that the serine/threonine protein phosphatase 2A (PP2A) is a key component in the regulation of stomatal movement under ALA-ABA treatments in apple [22] and *Arabidopsis* leaves [23]. PP2A holoenzymes typically exist as heterotrimers, including structural A, regulatory B, and catalytic C subunits [24, 25], which play critical roles in dephosphorylating proteins, thereby affecting kinase activity and cellular signal transduction [26]. In plants, PP2A is involved in regulating hormone-mediated signal transduction, including that of auxin [27, 28], ABA [29–31], ethylene [32, 33], methyl jasmonate (MeJA) [31, 34] and brassinosteroids (BRs) [35, 36]. Furthermore, different specific subunits of PP2A may play positive or negative roles in regulating ABA signal transduction [29, 37]. For instance, the ABA sensitivity of seed germination and stomatal movement was significantly impaired in the *pp2aa1* mutation of *Arabidopsis*, suggesting that *PP2AA1* functions positively in the regulation of ABA signal transduction [31]. Conversely, the *pp2ac2* mutant is more sensitive to ABA signals during seed germination, root growth, and seedling development, indicating that *PP2AC2* plays a negative role in regulation of ABA signal transduction [30]. Five genes encoding PP2A catalytic subunits (PP2AC) in *Arabidopsis* can be divided into subfamily I (*PP2A-C1*/*-C2*/*-C5*) and subfamily II (*PP2A-C3*/*-C4*) [38]. The genes in subfamily I may participate in plant defense responses to stress, because silencing of *PP2AC* in *Nicotiana benthamiana* leads to local cell death, suggesting that this catalytic subunit acts as a negative regulator of plant defense responses [39]. In potato and tomato, subfamily I genes are also involved in stress responses [40]. On the other hand, members of PP2AC subfamily II may participate in the process of auxin transport, because *pp2a-c3c4* double mutants show altered auxin distribution patterns [28]. To date, it remains unknown which subunits of PP2A are involved in the regulation of stomatal movement by ALA-ABA in apple leaves.

ABA is a critical factor in regulation of stomatal closure, and SRK2E/OST1/SnRK2.6 is an important component of the ABA signaling pathway [41–43]. Phosphorylated SnRK2.6 switches on downstream components of ABA signaling, while dephosphorylated SnRK2.6 is deactivated, thus ABA signals are no longer transduced and stomata will reopen [44, 45]. However, whether PP2A interacts with SnRK2.6 is unclear. We hypothesized that ALA might upregulate PP2A activity, in turn repressing SnRK2.6 activity to block ABA signaling and thus reversing ABA-induced stomatal closure.

Apple (*Malus* × *domestica* Borkh.) is the most common deciduous fruit tree cultivated in China, covering 2.088 million hectares in 2020 [46]. Not only is it a pillar of agricultural industry and a huge source of income, but, like all trees, it can absorb CO_2_ from the atmosphere and release O_2_ through photosynthesis, which has great ecological significance for mitigation of global warming. It has been estimated that an apple tree can absorb 700 g·CO_2_ m^−2^ annually [47]. If the valid leaf area index of an orchard is 2, then a hectare of fruit trees can fix 1400 kg CO_2_ in one year. The large number of apple trees in China will thus absorb about 3 million tons of CO_2_ from the atmosphere in a year, making apple orchards a significant carbon sink in the ecosystem. Furthermore, at the level of the individual leaf, the greater the stomatal opening, the more easily CO_2_ enters the mesophyll cells, which is fundamental for increased photosynthesis, high yields, and high quality. Unfortunately, environmental stresses often induce an increase in endogenous ABA, causing stomatal closure and a decrease in photosynthesis. ALA can significantly alleviate stress-induced stomatal closure and photosynthetic depression [2, 48]. Studying the regulatory mechanisms by which ALA affects stomatal movement by antagonizing ABA is therefore meaningful for horticultural science and practice.

In this study, we demonstrate that the interaction of MdPP2AC and SnRK2.6 plays a critical role in ALA-ABA regulation of stomatal movement. Our data showed that ALA significantly upregulated the expression of *MdPP2AC* and enhanced MdPP2AC protein abundance, MdPP2A enzyme activity, and MdPP2AC phosphorylation. At the same time, ALA suppressed the expression of *MdSnRK2.6* and its enzyme activity. Furthermore, MdPP2AC was shown to interact with MdSnRK2.6, leading to its dephosphorylation and thus inhibiting ABA-induced stomatal closure. The proposed signal transduction routes were verified in a series of transiently transgenic apple leaves, providing new insights into ALA regulation of stomatal movement in apple leaves.

## Results

### ALA and ABA regulate stomatal aperture and MdPP2A activity

**Figure 1:**
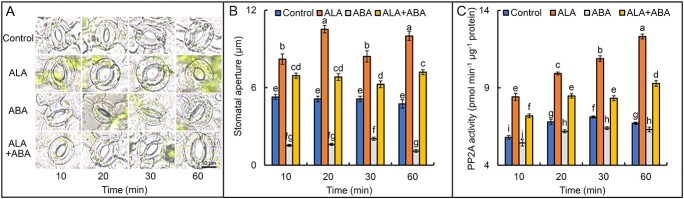
ALA and ABA regulate stomatal aperture in apple leaves, possibly via PP2A activity. **A:** Photographs of stomata after different treatments. **B:** Stomatal aperture after ALA and/or ABA treatment. Data are means of 40 measurements ± standard error (SE). **C:** PP2A activity in apple epidermal strips after different treatments. Data are from three independent biological replicates. The same lowercase letters in B or C indicate no significant difference at *p* = 0.05.

There were distinct differences in stomatal aperture among epidermal strips of apple leaves with different treatment for 10 min (Fig. 1A and B). ALA significantly promoted stomatal aperture and reversed ABA-induced stomatal closure. These effects were maintained for at least 60 min. The mean stomatal aperture of ALA-treated leaves was 83% greater than that of control leaves, whereas that of ABA-treated leaves was just 31% of the control. The stomatal aperture of leaves treated with ALA + ABA was 34% higher than that of control leaves, nearly 4 times as large as that of ABA treatment, suggesting that ALA eliminates ABA-induced stomatal closure.

Significantly, MdPP2A activity in the apple leaf epidermis was intensively affected by ALA and/or ABA treatment (Fig. 1C), and its variation trend was consistent with the stomatal opening (Fig. 1B). Pearson correlation analysis showed a high correlation coefficient of 0.838*** (Table S1) between MdPP2A activity and stomatal aperture, suggesting that MdPP2A activity may play an important role in ALA-ABA regulation of stomatal aperture in the apple leaf epidermis.

### ALA and ABA regulate *MdPP2A*-related gene expression

**Figure 2:**
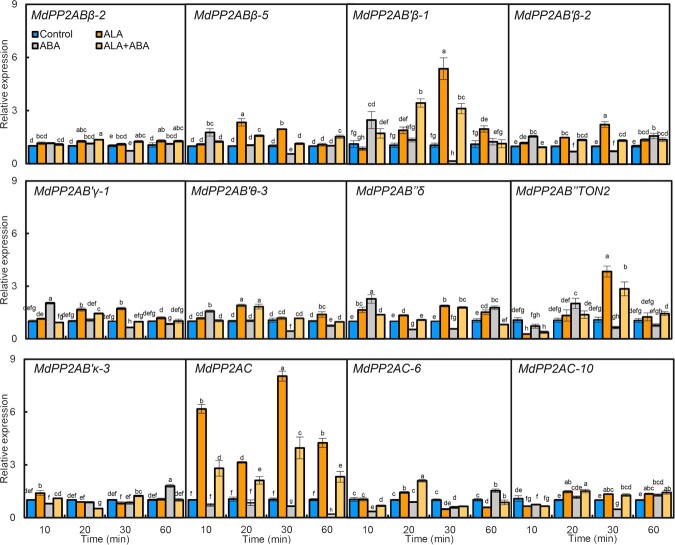
Expression of genes that encode parts of *PP2A* subunits in apple leaves treated by ALA or/and ABA**.** Epidermis preparation and treatments were as described in Fig. 1. The data are the means of three independent biological replicates. The same lowercase letters indicate no significant difference at *p* = 0.05.

According to the Apple Genome Database (https://www.ncbi.nlm.nih.gov/genome/?term=apple, *Malus domestica*: ASM211411v1), the apple *MdPP2A* family is consisted of 42 genes (Table S2). Their predicted proteins can be divided into three subfamilies based on comparison with their *Arabidopsis* counterparts: MdPP2AA scaffolding units, MdPP2AB regulatory units, and MdPP2AC catalytic subunits (Fig. S1). The expression levels of all 42 genes were measured by RT-qPCR in the apple leaf epidermis after ALA or/and ABA treatment, among which 12 changed significantly in response to different treatments at least once during the experiment are shown in Fig. 2; and others are shown in Fig. S2. Among the 12 selected genes, the expression of *MdPP2AC* showed the highest correlation with changes of stomatal aperture and MdPP2A activity, with correlation coefficients of 0.724^***^ and 0.758^***^ (Table S2), respectively. This indicates that *MdPP2AC* may play an important role in ALA and ABA-regulated stomatal movement.

### ALA enhances MdPP2AC protein abundance and phosphorylation

**Figure 3:**
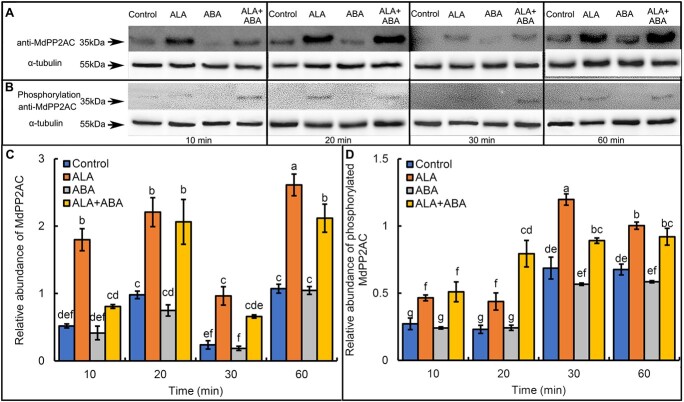
ALA significantly increases abundance and phosphorylation of the MdPP2AC protein. **A:** ALA promotes MdPP2AC abundance when α-tubulin (actin) was used as loading control. Molecular mass (kDa) markers are shown on the left. All experiments were repeated at least three times with similar results. **B:** Total plant proteins were extracted and immunoprecipitated with a phosphorylated MdPP2AC antibody. Image J software was used to estimate the relative abundance of total (**C**) and phosphorylated (**D**) MdPP2AC protein. Data are means of three independent biological replicates. The same lowercase letters in **C** or **D** indicate no significant difference at *p* = 0.05.

To determine whether ALA and ABA regulate MdPP2AC at the protein level, a customized MdPP2AC-specific antibody (Appendix 3) was used to detect abundance of the MdPP2AC protein. Western blotting analysis showed that ALA significantly upregulated MdPP2AC abundance, but ABA itself had no significant effect on MdPP2AC abundance (Fig. 3A and C). In addition, when a commercial phosphorylated PP2AC antibody (Appendix 4) was used, ALA significantly promoted MdPP2AC phosphorylation, whereas ABA once again had no significant effect (Fig. 3B and D). Thus, only ALA affected the abundance and phosphorylation level of the MdPP2AC. By contrast, ABA had no such effect on MdPP2AC abundance or phosphorylation.

### Interactions of MdPP2AC with the other subunits and their promotion by ALA

Although MdPP2AC plays an important role in the regulation of stomatal movement by ALA-ABA in apple leaves, there are numerous subunits in the PP2A holoenzyme. For this reason, we decided to study the interactions of MdPP2AC with the other subunits. In yeast two-hybrid (Y2H) assays, MdPP2AC was found to interact with MdPP2AA, MdPP2AAβ, MdPP2ABβ-2, MdPP2AB’β-2, MdPP2AB’γ-1, and MdPP2AC-10 (Fig. S3A) but not with MdPP2AAβ-1, MdPP2AAβ-2, MdPP2ABβ-5, MdPP2AB’β-1, MdPP2AB’θ-3, MdPP2AB’κ-3, MdPP2AB”TON2, MdPP2AB”δ, or MdPP2AC-6 (Fig. S4). These suggest that MdPP2AC can interact with parts of the A, B, and C subunits of the holoenzymes. The interactions observed in Y2H assays were subsequently verified by BiFC and FLC assays (Fig. S3B and C).

**Figure 4:**
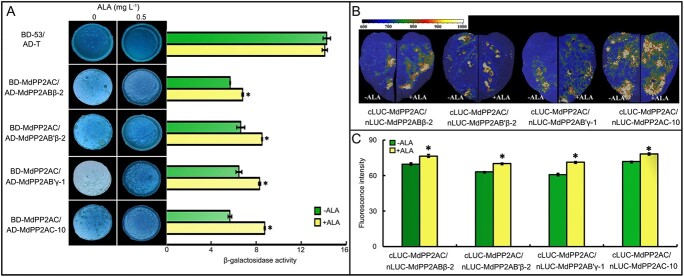
ALA promotes the interaction of MdPP2AC with other MdPP2A subunits**. A:** Quadruple dropout medium (SD/−Ade/–His/−Leu/−Trp supplemented with X-α-Gal and AbA) with 0.5 mg L^−1^ ALA was used in Y2H assays to test potential interactions. BD-53 plus AD-T was applied for the positive control. The β-galactosidase activity was detected by ONPG (o-nitrophenyl β-D-galactopyranoside) assay. **B:** FLC assays. **C:** Intensity of the luciferase interaction signal was measured with ImageJ software. Values are means ± SE from three independent experiments (^*^p < 0.05).

Addition of ALA significantly promoted the protein interactions as measured by β-galactosidase activity in yeast and fluorescence intensity in tobacco leaves (Fig. 4), suggesting that ALA promotes interactions between PP2AC and other subunits of PP2A holoenzymes.

### ALA impairs ABA-induced *MdSnRK2.6* expression and MdSnRK2.6 phosphorylation

**Figure 5:**
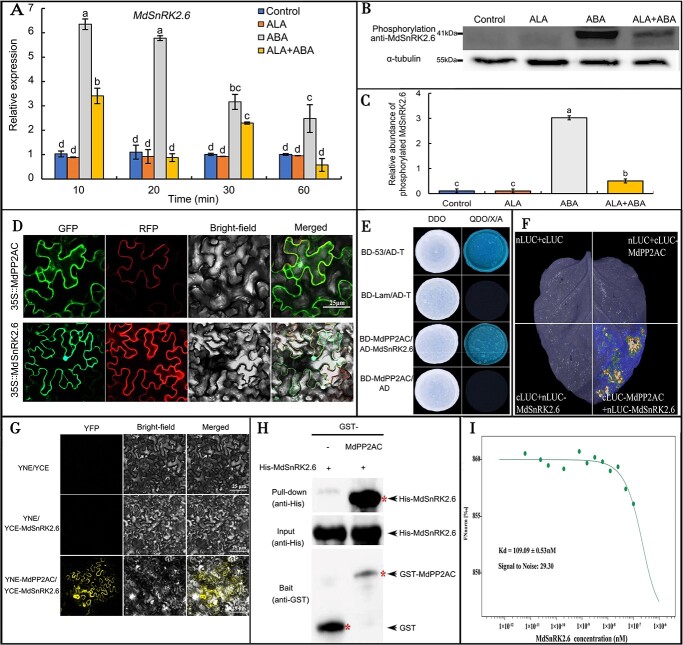
ALA inhibits the ABA-induced expression of *MdSnRK2.6* and promotes MdSnRK2.6 dephosphorylation, and MdSnRK2.6 interacts with MdPP2AC**. A:** Expression of *MdSnRK2.6* in apple leaves under ALA and/or ABA treatment. **B:** Western blotting assay of phosphorylated MdSnRK2.6, whose abundance was specifically induced by ABA but reduced by ALA. The total proteins of apple leaf epidermis under different treatments were extracted before immunoprecipitation with a specific phosphorylated SnRK2.6 antibody. **C:** Quantification of the phosphorylated SnRK2.6 levels in **B**. The same lowercase letters in **A** or **C** indicate no significant difference at *p* = 0.05. **D:** The subcellular localization of MdPP2AC and MdSnRK2.6 in tobacco leaves. The pCAMBIA1305-mCherry membrane marker and pCAMBIA1300-35S::*GFP*, 35S::*MdPP2AC*, and 35S:: *MdSnRK2.6* recombinant plasmids were transiently co-expressed in 4-week-old *N. benthamiana* leaves. RFP: Red fluorescent protein. GFP: green fluorescent protein. Scale bar: 25 μm. **E:** A Y2H assay confirmed the interaction of MdPP2AC with MdSnRK2.6. The combination of BD-53 plus AD-T was used as a positive control, and BD-Lam plus AD-T was used as a negative control. **F:** The interaction of MdPP2AC and MdSnRK2.6 showed by an FLC assay. **G:** A BiFC assay showed the interaction of MdPP2AC and MdSnRK2.6. **H:** Pull-down assay. MdSnRK2.6-His and MdPP2AC-GST proteins were expressed in *E. coli* for immunoprecipitation. The anti-His and anti-GST antibody were used for detection of MdSnRK2.6-His and MdPP2AC-GST, respectively. **I:** Interaction between MdPP2AC and MdSnRK2.6 measured by MST. Despite small changes in the mass of MdPP2AC, a signal-to-noise ratio of 29.30 enabled accurate determination of the interaction strength, and the affinity between MdPP2AC and MdSnRK2.6 was determined to be 109.09 ± 0.53 nM. Three independent experiments were performed with similar results.

SnRK2.6 plays a key role in ABA-induced stomatal closure [41, 43, 49]. However, the relationship between MdSnRK2.6 and ALA signals has not been reported. Here, we measured *MdSnRK2.6* expression after different treatments and found that ALA itself did not affect *MdSnRK2.6* expression, whereas ABA rapidly stimulated its expression. However, ALA impaired the ABA-induced expression of *MdSnRK2.6* (Fig. 5A), suggesting that cross-talk between ALA and ABA may occur in the regulation of *MdSnRK2.6* expression.

To detect whether ALA could affect MdSnRK2.6 phosphorylation, we used an *Arabidopsis* antibody for phosphorylated AtSnRK2.6 [50] to detect the levels of MdSnRK2.6 phosphorylation in apple leaves after different treatments. No phosphorylated MdSnRK2.6 was detected in the control or ALA-treated leaves, but a phosphorylated MdSnRK2.6 protein band clearly appeared after ABA treatment and was significantly inhibited by exogenous ALA (Fig. 5B and C). These results suggest that the phosphorylated AtSnRK2.6 antibody can recognize phosphorylated MdSnRK2.6 of apple and that MdSnRK2.6 phosphorylation is specifically induced by ABA but reversed by ALA.

### Detection of the MdPP2AC–MdSnRK2.6 interaction

Because both PP2AC and SnRK2.6 participate in ALA-ABA regulated stomatal movement, we next asked whether the two proteins interacted. First, we analyzed their subcellular locations. MdPP2AC was localized in the plasmalemma and cytoplasm, whereas MdSnRK2.6 was present in the plasmalemma, cytoplasm, and nuclei (Fig. 5D). We next performed a series of Y2H, FLC, BiFC, and pull-down assays, as well as a microscale thermophoresis (MST) experiment. The results of all these assays demonstrated an interaction between MdPP2AC and MdSnRK2.6 (Fig. 5E-I). We therefore speculated that MdPP2AC may interact and dephosphorylate MdSnRK2.6, thereby blocking ABA-induced stomatal closure.

### Gene manipulation of *MdPP2AC* or *MdSnRK2.6* and ALA treatment affect stomatal movement in transiently transgenic apple leaves

**Figure 6:**
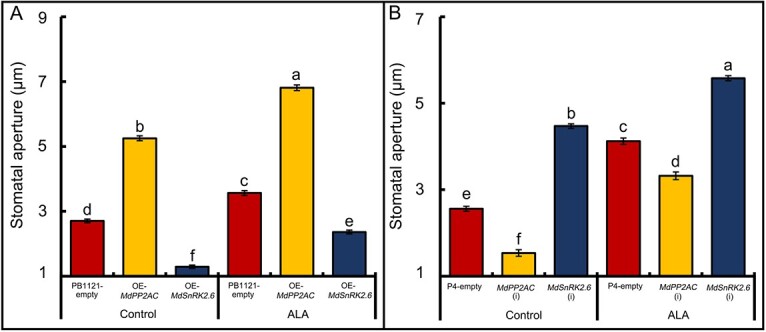
ALA treatment affects stomatal movement in transiently transgenic apple leaves**. A**: Stomatal aperture of apple leaves transiently overexpressing *MdPP2AC* or *MdSnRK2.6* and treated with or without ALA. **B**: Stomatal aperture of apple leaves in which *MdPP2AC* or *MdSnRK2.6* was transiently silenced, treated with or without ALA. Values are means ± SE of three biological replicates, and the same lowercase letters indicate no significant difference at *p* = 0.05.

We generated transiently transgenic apple leaves in which the genes were overexpressed or partially silenced to verify the functions of MdPP2AC and MdSnRK2.6 in stomatal movement. The OE-MdPP2AC and OE-MdSnRK2.6 transformations were detected by GUS staining (Fig. S5), and expression of the transgenes was measured by RT-qPCR (Fig. S6), confirming that the target genes had been transformed. Stomatal aperture measurements showed that OE-*MdPP2AC* promoted stomatal opening, which was further promoted by exogenous ALA treatment (Fig. 6, Fig. S7). On the other hand, OE-*MdSnRK2.6* led to stomatal closure, although ALA still promoted stomatal opening (Fig. 6). Conversely, *MdPP2AC* (i) induced stomatal closure, whereas *MdSnRK2.6* (i) increased stomatal aperture. The stomatal aperture of leaf epidermal strips increased in all genotypes under exogenous ALA treatment (Fig. 6, Fig. S7), indicating that regardless of overexpression or interference of these genes, ALA can promote stomatal opening.

### Gene manipulation of *MdPP2AC* or *MdSnRK2.6* and ALA treatment affect MdPP2A and MdSnRK2.6 activities in transgenic apple leaves

**Figure 7:**
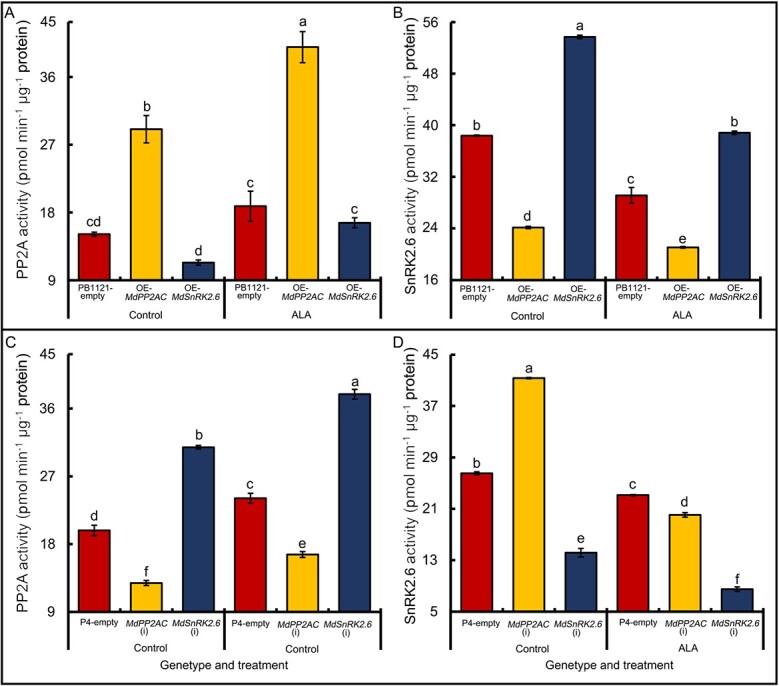
Effects of ALA on MdPP2A (A, C) and MdSnRK2.6 (B, D) activities in the leaf epidermis of different transiently transgenic apple genotypes**.** Values are means ± SE of three biological replicates. The same lowercase letters indicate no significant difference at *p* = 0.05.

The MdPP2A activity in transiently transgenic OE-*MdPP2AC* apple leaves was significantly increased (Fig. 7A). Conversely, MdPP2A activity was significantly reduced in partially *MdPP2AC* silenced leaves (Fig. 7C). Enzyme activities increased in both genotypes under ALA treatment. These results suggest that OE-*MdPP2AC* promotes PP2A activity, which is further promoted by exogenous ALA. When *MdSnRK2.6* was overexpressed, PP2A activity was not affected, but ALA promoted the PP2A activity in OE-*MdSnRK2.6* (Fig. 7A), suggesting that MdSnRK2.6 acts downstream of MdPP2A, whereas ALA acts upstream. When *MdSnRK2.6* expression was partially silenced, PP2A activity increased significantly, and this effect was further stimulated by exogenous ALA (Fig. 7C). These phenomena also suggest that MdSnRK2.6 acts downstream of MdPP2A, and the former may have a feedback regulatory effect on the latter.

Measurements of MdSnRK2.6 activity also revealed the possible mechanism. When *MdPP2AC* was overexpressed, SnRK2.6 activity was significantly reduced, and exogenous ALA further aggravated this effect (Fig. 7B). When the expression of *MdPP2AC* was partially silenced, SnRK2.6 activity increased significantly, but exogenous ALA could reduce the activity (Fig. 7D). When *MdSnRK2.6* was overexpressed, SnRK2.6 activity was significantly increased, and exogenous ALA inhibited this effect (Fig. 7B). When expression of *MdSnRK2.6* was partially silenced, SnRK2.6 activity was significantly reduced, and exogenous ALA further lowered this activity (Fig. 7D). Therefore, MdSnRK2.6, acting downstream of PP2A signaling, may provide feedback to regulate PP2A activity, suggesting that these regulatory mechanisms are very complex. In any event, there was an extremely significant negative Pearson’s correlation coefficient between PP2A and SnRK2.6 activities (−0.789^***^) in these transgenic apple leaves.

**Figure 8:**
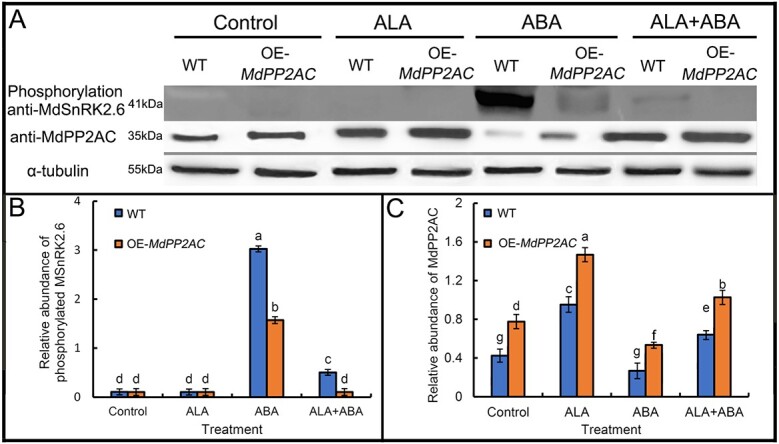
Western blotting assay of phosphorylated MdSnRK2.6 and MdPP2AC protein abundances in OE-*MdPP2AC* apple leaves treated with exogenous ALA and/or ABA**. A**: Western blotting assay. **B and C**: Quantification of grey levels of the phosphorylated SnRK2.6 and PP2AC protein bands. Proteins were extracted from the apple leaf epidermis under different treatments before immunoprecipitation. Molecular mass (kDa) markers are shown on the left. All experiments were repeated at least three times with similar results. The same lowercase letters indicate no significant difference at *p* = 0.05

To verify the induction of MdSnRK2.6 dephosphorylation by MdPP2AC, we exposed OE-*MdPP2AC* transiently transgenic apple leaves to ALA and/or ABA treatment, then analyzed their extracted proteins by western blotting with the *Arabidopsis* phosphorylated SnRK2.6 antibody (Fig. 8). Phosphorylated MdSnRK2.6 was detected only when ABA was present in either WT or transgenic leaves. Compared with the WT, the OE-*MdPP2AC* transgenic leaves contained less phosphorylated MdSnRK2.6 after ABA treatment. When both ALA and ABA were applied, the abundance of phosphorylated MdSnRK2.6 decreased further in both genotypes. Here again, we demonstrated that *MdPP2AC* overexpression inhibits MdSnRK2.6 phosphorylation and the upregulation of MdPP2AC by exogenous ALA can promote dephosphorylation of the phosphorylated MdSnRK2.6 induced by ABA.

### Gene manipulation of *MdPP2AC* or *MdSnRK2.6* and ALA treatment affect Ca^2+^, H_2_O_2_, and flavonol levels in guard cells of transgenic apple leaves

**Figure 9:**
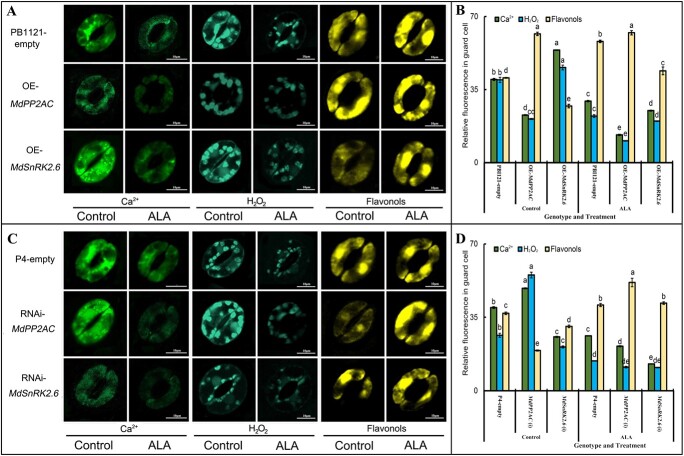
Effects of ALA treatment on Ca^2+^, H_2_O_2_, and flavonol levels in stomatal guard cells of different genotypes of transiently transgenic apple leaves**. A** and **C:** Photographs of Ca^2+^, H_2_O_2_, and flavonol levels in stomatal guard cells. Scale bar: 10 μm. **B** and **D:** Relative fluorescence intensity of Fluo-3 a.m., H_2_DCF-DA, and DPBA in guard cells. Each treatment was measured with ImageJ software. Values are means ± SE of 15 replicate measurements. Bars that share lowercase letters are not significantly different at *p* = 0.05.

Ca^2+^, H_2_O_2_, and flavonols are the key components in regulation of stomatal movement. The content of Ca^2+^ and H_2_O_2_ was significantly lower in guard cells of OE-*MdPP2AC* transgenic leaves, whereas the flavonol content was markedly higher. Conversely, guard cells of OE-*MdSnRK2.6* had much more Ca^2+^ and H_2_O_2_ and less flavonols than PBI121-empty (Fig. 9A and B). These results suggest that overexpression of *MdPP2AC* can decrease Ca^2+^ and H_2_O_2_ levels and promote flavonol accumulation in guard cells, whereas overexpression of *MdSnRK2.6* has the opposite effects. When *MdPP2AC* expression was partially silenced, Ca^2+^ and H_2_O_2_ levels in guard cells increased, and flavonol levels decreased; the opposite result was observed with partial silencing of *MdSnRK2* (Fig. 9C and D). Nonetheless, ALA promoted flavonol accumulation and reduced Ca^2+^ and H_2_O_2_ levels in all transgenic plants, suggesting that ALA acts at an upstream position whose functions influence all the tested genes.

## Discussion

Since Hotta *et al.* (1997) [5] reported that ALA promoted plant growth and increased crop yield, the multiple bioregulatory functions of this non-protein amino acid have been gradually revealed. In our laboratory, ALA has been studied for 20 years (Table S3) [2, 4]. The basic biological role of ALA is as an essential precursor for chlorophyll biosynthesis [1], which is necessary for light quantum harvesting and photosynthetic energy transfer [51]. Because photosynthesis and photosynthate accumulation are promoted by ALA, soluble sugars are significantly increased in many fruit species, such as apple [52–54], grape [55, 56], kiwi [57], and tomato [58], and the fruit flavor quality is improved. ALA can also improve anthocyanin accumulation in apple [13–16], peach [59], litchi [60], and grape [61], thus improving external fruit quality. ALA can also promote flavonol accumulation in apples [15, 17, 54], which is beneficial for human health [62]. In addition, ALA has been reported to promote carotenoid biosynthesis and improve nutritional quality in tomato [63]. One of the more interesting functions of ALA is to improve plant tolerance to abiotic and biotic stresses [6, 10] such as chilling [64], salinity [3], heat [65], strong light [66], UV-B light [67], low light [68], drought [11], waterlogging [7], alkaline soil [69], nitrogen deficiency [70], urban roadside air pollution [71], heavy metal pollution [72], pesticides [73], herbicide damage [74], and fungal infection [75]. In addition to research on exogenous ALA application, studies on transgenic plants that overproduce endogenous ALA have confirmed its biological functions. When *Yhem1* (a yeast *Hem1* driven by the *Arabidopsis HemA* promoter, which is light-inducible) was transformed into higher plants, transgenic tobacco [76, 77], strawberry [78], tomato [79], and canola [80] biosynthesized much more endogenous ALA and chlorophyll and had higher photosynthetic rates and energy conversion efficiency than their respective WTs. *Yhem1* transgenic Arabidopsis [81], tomato [82], and canola [83] have been demonstrated to be more salt tolerant, and transgenic banana was colder tolerant [84]. In our study, we detected the endogenous ALA content in leaf epidermis of PEG-stressed apple plants. The results showed that water stress induced much more ALA accumulation in the epidermis (Fig. S9), which suggests that ALA may have important biological significance. In addition, the stomatal conductance of the transgenic plants was significantly higher than that of the wild type [76, 77], where ABA-induced stomatal closure was impaired [19, 20]. Therefore, the regulatory function of ALA in inducing stress tolerance is similar but not identical to that of ABA. In fact, ALA antagonizes the effect of ABA on stomatal movement of plant leaves [18, 20]. When ALA-pretreated strawberries were stressed with PEG-6000, they could tolerate osmotic stress while showing greater stomatal conductance, net photosynthetic rates, biomass, and water balance than controls [11]. This result suggests that ALA improves drought tolerance while simultaneously increasing stomatal aperture [20].

Stomata are the main gateway for CO_2_ uptake, directly affecting intercellular CO_2_ levels and leaf photosynthesis [85]. Environmental stresses frequently induce endogenous ABA accumulation, leading to stomatal closure, which in turn hinders CO_2_ diffusion into the leaf mesophyll and decreases photosynthesis. Conversely, ALA antagonizes ABA- or PEG-induced stomatal closure [11, 18]. It has been reported that mean net photosynthetic rate and stomatal conductance of pear leaves after a full-day ALA treatment were about 14% and 21% higher than those of control leaves [86]. These findings suggest that ALA treatment enables plants to absorb more atmospheric CO_2_ and synthesize more photosynthates. Nevertheless, the underlying mechanisms by which ALA influences stomatal movement have not been thoroughly elucidated.

In recent years, our research group has focused on the regulation of leaf stomatal movement by ALA and ABA [18–23, 87, 88]. In the present study, we again observed that ALA inhibited ABA-induced stomatal closure in the epidermis of apple leaves (Fig. 1A and B). Moreover, stomatal movement was highly correlated with MdPP2A activity (Fig. 1C), consistent with the previous report [22], who found that the PP2A inhibitor okadaic acid aggravated ABA-induced stomatal closure in apple leaves and that ALA effectively reversed the effect of ABA. An *et al*. (2020) [23] reported that overexpression of *AtPP2A-C2* promoted ALA-ABA-regulated stomatal opening, whereas the *atpp2a-c2* mutant had a reduced stomatal aperture. Therefore, PP2AC is an important positive component in ALA-reversable, ABA-induced stomatal closure in *Arabidopsis*. In this study, we analyzed expression levels of all apple genes encoding subunits of the MdPP2A holoenzyme by RT-qPCR after ALA and/or ABA treatments. We found that expression of some genes was highly correlated with stomatal aperture and PP2A activity (Fig. 2 and Fig. S2), the most significant correlation was observed for *MdPP2AC* (Table S1). These results were consistent with previous observations in *Arabidopsis* [23]. Pernas *et al*. (2007) [30] proposed that AtPP2AC might act as a negative regulator in the ABA signaling pathway. However, we did not find that ABA significantly affected MdPP2AC abundance in the epidermis of apple leaves (Fig. 3). In fact, only ALA had actual effects on *MdPP2AC* expression (Fig. 2), protein abundance and phosphorylation (Fig. 3), and enzyme activity (Fig. 1). All these changes are involved in ALA-induced stomatal opening. Pernas *et al*. (2007) [30] offered the caveat that rapid decreases in PP2A activity upon ABA treatment might not be reliable, as the data fluctuated greatly. Therefore, we suspect that ABA signaling may act downstream of the ALA signaling pathway beyond PP2AC and that ALA rather than ABA affects PP2A activity at different levels. In addition, we found that MdPP2AC interacted with other PP2A subunits, such as MdPP2AA, MdPP2AAβ, MdPP2ABβ-2, MdPP2AB’β-2, MdPP2AB’γ-1, and MdPP2AC-10 (Fig. S3), and these interactions were promoted by exogenous ALA (Fig. 4). These results suggest that ALA may promote PP2A holoenzyme assembly and increase its enzyme activity.

**Figure 10:**
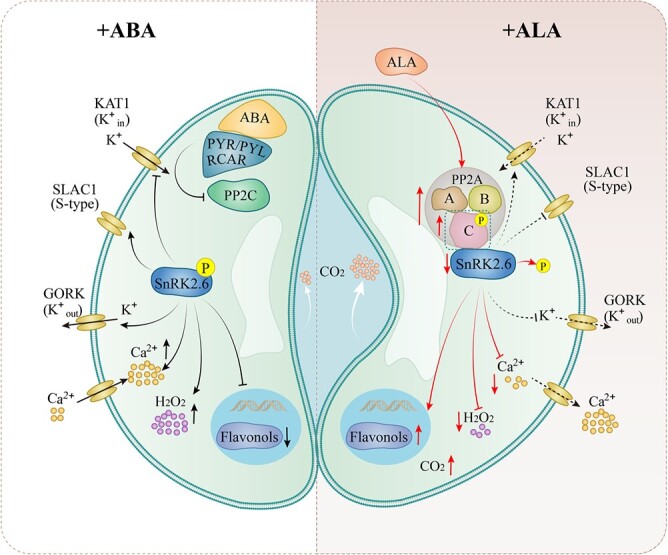
A proposed model for the reversal of ABA-induced stomatal closure by ALA**.** → represents a positive effect, and —| represents a negative effect, − - - - represents speculation. Processes in red are studied in the present work, and those in black have been reported previously.

The regulation of the PP2A holoenzyme itself is complex [89, 90]. In *Arabidopsis*, scaffolding subunit A and catalytic subunit C comprise a core enzyme that then binds to different regulatory B subunits to form a variety of heterotrimeric complexes [91]. Distinct regulatory subunits determine substrate specificity, subcellular localization, and catalytic activity of the PP2A holoenzyme [26, 92]. In the apple genome, 42 genes encode different subunits of MdPP2A holoenzymes and can be divided into three groups, A, B, and C (Fig. S1), which are homologous to their counterparts in *Arabidopsis* [24]. Among the apple genes, 4 encode scaffolding subunit A, 28 encode regulatory subunit B, and 10 encode catalytic subunit C (Fig. S1 and Table S3). In *Arabidopsis*, ABA-induced stomatal closure is impaired in *rcn1* mutants, implying that *RCN1*, which encodes the AtPP2AA subunit, is involved in ABA-induced stomatal closure [31]. In our study, ALA downregulated *MdPP2AA* expression (Fig. S2), and the MdPP2AA protein interacted with MdPP2AC (Fig. S3). However, *MdPP2AA* expression was not significantly negatively correlated with stomatal aperture (Table S2), suggesting that it is not a key component in ALA-induced stomatal opening in apple. Since MdPP2AC is a catalytic and it responded to ALA more significantly, we paid more attention to it in the present study. Measurements of MdPP2AC protein abundance and phosphorylation (Fig. 3), and molecular interactions (Fig. S3, Fig. 4) demonstrated that the catalytic subunit MdPP2AC plays a core role in the signaling pathway of ALA-induced stomatal opening. This conclusion was further verified by transient overexpression and partial silencing of *MdPP2AC* in transgenic apple leaves (Fig. 6). In *Arabidopsis* roots, AtPP2AC3 or AtPP2AC4 interacts with and dephosphorylates the PIN kinase to affect auxin transport [93]. Here, Y2H, BiFC, FLC, pull-down, and MST assays all showed that MdPP2AC interacts with MdSnRK2.6 (Fig. 5). Overexpression and partial silencing of *MdPP2AC* in transiently transgenic apple leaves had negative and positive effects, respectively, on SnRK2.6 activity (Fig. 7). These results suggest that MdPP2AC is located upstream of the signaling pathway, negatively regulating MdSnRK2.6 activity to downregulate ABA signaling and promote stomatal reopening.

SnRK2.6 is well known as a critical component of the ABA signaling pathway [94], and it can be activated within minutes of exogenous ABA application. In *Arabidopsis*, OST1/SnRK2.6 interacts with PP2AA subunits such as A and A3, B9α, B9β, and B9δ, but not with C3 [94]. In the present study, ABA rapidly upregulated *MdSnRK2.6* expression several fold and promoted stomatal closure of apple leaves within 10 min (Fig. 5). MdSnRK2.6 activity was significantly and negatively correlated with stomatal aperture (r = −0.660^**^) and MdPP2A activity (r = −0.595^*^) (Table S1). Furthermore, we found that MdSnRK2.6 phosphorylation was significantly induced by ABA, and this effect was significantly inhibited by ALA treatment (Fig. 5). Our western blotting was performed using an *Arabidopsis* antibody to AtSnRK2.6. This antibody was prepared with the peptide SVLHSQPK-pS-TVGTP-amide as the antigen [50], and this sequence is completely homologous to the amino acid sequence of apple MdSnRK2.6. Therefore, the antibody can recognize the apple phosphorylated site to assess abundance of phosphorylated MdSnRK2.6. However, NCBI blast results indicated that the peptide sequence is also homologous to that of MdSnRK2.2/3/7, and the antibody might recognize the other MdSnRK2s. However, only SnRK2.6 phosphorylation is induced by ABA [50], and the phosphorylated protein detected here should be MdSnRK2.6, rather than other MdSnRK2s. In the OE-*MdPP2AC* transiently transgenic leaves, ABA-induced MdSnRK2.6 phosphorylation was clearly suppressed, and exogenous ALA promoted further dephosphorylation (Fig. 8). These results suggest that MdPP2AC may interact with MdSnRK2.6 to catalyze its dephosphorylation and inactivate its kinase activity.

In ABA signaling pathway, SnRK2.6 phosphorylation is necessary for ABA-induced stomatal closure [49]. In the present study, stomatal aperture was significantly reduced when *MdSnRK2.6* was transiently transformed into apple leaves and increased when *MdSnRK2.6* was partially silenced (Fig. 6). Exogenous ALA significantly decreased SnRK2.6 enzyme activity (Fig. 7), resulting in greater stomatal aperture (Fig. 6). Therefore, we propose that ALA reverses ABA-induced stomatal closure by downregulating SnRK2.6 kinase activity through protein dephosphorylation via phosphorylated PP2AC. Yu *et al*. [ 95] proposed that AtPP2A dephosphorylates ABI5, a bZIP transcription factor, to stop ABA signal transduction. MdPP2AC can interact with MdSnRK2.6 (Fig. 5), causing the latter’s dephosphorylation (Fig. 8), suggesting that a similar mechanism may occur during ALA-ABA regulation of stomatal movement in apple leaves.

Calcium ions and H_2_O_2_ are involved in stomatal regulation [49] and have been found to participate in ALA-ABA-regulated stomatal movement in apple [18, 21] and *Arabidopsis* [20]. Furthermore, ALA promotion of stomatal opening depends on flavonol accumulation in the guard cells [21]. In this study, we found that *MdPP2AC* overexpression significantly increased flavonol content while reducing Ca^2+^ and H_2_O_2_ levels in guard cells of transiently transgenic apple leaves, whereas *MdSnRK2.6* overexpression showed the opposite effect (Fig. 9). When the expression of *MdPP2AC* was partially silenced, Ca^2+^ and H_2_O_2_ levels increased in the guard cells, and flavonols decreased. The opposite result was observed when *MdSnRK2.6* was partially silenced. Exogenous ALA decreased Ca^2+^ and H_2_O_2_ but increased flavonols in all plant genotypes (Fig. 9), which were highly correlated with stomatal aperture. Thus, Ca^2+^, H_2_O_2_, and flavonols act downstream of the PP2AC and SnRK2.6 signaling pathway during ALA-ABA regulation of stomatal movement in apple leaves.

Based on the current study and previous reports, we propose a working model for the antagonism of ABA signaling by ALA at SnRK2.6 to promote stomatal opening (Fig. 10). When ABA is present, it interacts with the PYR/PYL/RCAR receptors, binding PP2C and releasing SnRK2.6, which is then phosphorylated and activated, and in turn promotes Ca^2+^ and H_2_O_2_ increases and reduces flavonols in the guard cells. Then, KAT1 (K^+^_in_ channel) are blocked, whereas SLAC1 (slow anion channel-associated 1) and GORK (K^+^_out_ channel) opening are promoted. By contrast, when ALA is present, it upregulates *MdPP2AC* expression, increasing MdPP2AC abundance and phosphorylation, promoting the interactions of different PP2A subunits, and increasing holoenzyme activity. Phosphorylated PP2A interacts with and dephosphorylates MdSnRK2.6, leading to reduce cytoplasmic Ca^2+^ and H_2_O_2_ but rise flavonols and consequently, K^+^ and water flow into the guard cells to open stomata. In this model, MdSnRK2.6 is the key point where ALA-ABA crosstalk occurs; ABA signaling leads to MdSnRK2.6 phosphorylation, followed by stomatal closure, whereas ALA signaling causes its dephosphorylation, followed by stomatal opening. More CO_2_ then enters the mesophyll cells, even under stress conditions [96], which is necessary for photosynthesis and carbon fixation.

## Conclusions

Most previous studies on ALA-induced stomatal opening have been based on pharmacological physiological experiments. This is the first time in which multiple molecular biological techniques have been used to explore the mechanisms by which ALA promotes stomatal opening. We confirmed that MdPP2A activity is critical for the ability of ALA to reverse ABA-induced stomatal closure. Among 42 genes encoding different subunits of the MdPP2A holoenzyme, *MdPP2AC* showed the strongest response to ALA treatment. Exogenous ALA can promote multiple aspects of *MdPP2AC*, such as gene expression, protein translation and phosphorylation, and interactions with other subunits. We also found that MdPP2AC can interact with and dephosphorylate MdSnRK2.6, negatively regulating ABA signaling and altering Ca^2+^, H_2_O_2_, and flavonol levels in guard cells, thus leading to stomatal reopening. This hypothesis was verified in a series of transiently transgenic apple leaves. The roles of other PP2A subunits and genes remain to be clarified. Many of them showed strong responses to ALA treatment, suggesting that they may also be involved in stomatal regulation.

## Materials and methods

### Plant materials and chemical treatments

Test-tube apple (*Malus* × *domestica* Borkh. cv. Fuji) plantlets were cultured and chemically treated as previously described [97].

### Measurement of stomatal aperture

Stomatal aperture after different treatments were determined according to Chen and Wang (2022) [97].

### P‌P2A and SnRK2.6 activity assays

The total protein of epidermal strips from apple leaves were extracted as previously described [97]. PP2A and SnRK2.6 enzyme activities were determined using the plant protein phosphatase (PP2A) enzyme-linked immunoassay kit (Shanghai Fusheng Industrial Co., Ltd., Item No. A024850-96 T, Appendix 1) and an SnRK2.6 enzyme-linked immunoassay kit (Item No. A089115-96 T, Appendix 2), respectively. Three independent biological replicates were performed for each experiment.

### Identification of PP2A in apple and *Arabidopsis* genomes

We used the Hidden Markov Model (HMM) [98] and the Simple Modular Architecture Research Tool (SMART) [99] to identify PP2A genes within the apple genome (https://www.ncbi.nlm.nih.gov/genome/?term=apple, *M. domestica*: ASM211411v1) and *Arabidopsis* genome (http://www.ncbi.nlm.nih.gov/genome/?term=Arabidopsis+thaliana, assembly TAIR10.1). The Pfam number of the protein sequence was obtained from the Pfam protein family database (http://pfam.xfam.org/) by downloading the relevant HMM profile.

### Subcellular localization of MdPP2AC and MdSnRK2.6 in *N. benthamiana*

The genes *MdPP2AC* and *MdSnRK2.6* without stop codons were ligated into the pCambia1300 vector. The recombinant plasmids pCambia1300-*MdPP2AC*-GFP and pCambia1300-*MdSnRK2.6*-GFP were then transformed into *GV3101* competent cells (Shanghai Weidi Biotechnology Co., Ltd, CAT#: AC1001). To determine whether MdPP2AC and MdSnRK2.6 were present in the plasma membrane, pCAMBIA1300-35S::*MdPP2AC*-GFP and pCAMBIA1300-35S::*MdSnRK2.6*-GFP were co-expressed with the membrane marker protein pCAMBIA1305-*mCherry* via *Agrobacterium tumefaciens*–mediated transformation in *N. benthamiana*. An ultra-high-resolution laser confocal microscope (LSM 800, Zeiss, Germany) was used to observe fluorescence 48 h after infection. All the primer sequences used in this study were listed in Table S4.

### RNA isolation and RT-qPCR

RNA isolation and RT-qPCR were performed as previously described [97]. The 2^−ΔΔCT^ method was used to calculate relative gene expression [100].

### Yeast two-hybrid assay and measurement of β-galactosidase activity

*MdPP2AC* was ligated into the pGBKT7 vector (Clontech) as bait. The full-length sequences of *MdPP2AA*, *MdPP2AAβ*, *MdPP2AAβ-1*, *MdPP2AAβ-2*, *MdPP2ABβ-2*, *MdPP2ABβ-5*, *MdPP2AB’β-1*, *MdPP2AB’β-2*, *MdPP2AB’γ-1*, *MdPP2AB’θ-3*, *MdPP2AB′′TON2*, *MdPP2AB′′δ*, *MdPP2AC-6*, *MdPP2AC-10*, and *MdSnRK2.6* were ligated into the pGADT7 vector as prey. Prey and bait plasmids were mated and transformed into the yeast strain Y2HGold (Clontech, USA). To determine whether ALA could promote interactions between MdPP2AC and MdPP2ABβ-2, MdPP2AB’β-2, MdPP2AB’γ-1, and MdPP2AC-10, an assay kit (Shanghai Fusheng Industrial Co., Ltd., Item No. A087910-96 T) was used to measure β-galactosidase activity.

### Bimolecular fluorescence complementation assay

The cDNA of *MdPP2AC* without the stop codon was ligated into pSPYCE173 [101] via the BamHI and XhoI sites to obtain SYFP-N-*MdPP2AC*. The cDNAs of *MdPP2AA*, *MdPP2AAβ*, *MdPP2ABβ-2*, *MdPP2AB’β-2*, *MdPP2AB’γ-1*, *MdPP2AC-10*, and *MdSnRK2.6* without stop codons were amplified and cloned into the BamHI and XhoI sites of pSPYCE155 to construct the SYFP-C-*MdPP2AA*, SYFP-C-*MdPP2AAβ*, SYFP-C-*MdPP2Abβ-1*, SYFP-C-*MdPP2Abβ-2*, and SYFP-C-*MdSnRK2.6* plasmids. The transformation of recombinant plasmid into *A. tumefaciens GV3101* infecting *N. benthamiana* plants and the observation of fluorescence signal were referred to Chen and Wang (2022) [97].

### Firefly luciferase complementation imaging assay

The *MdPP2AC* removed stop codon was fused to the pC1300-nLUC (luciferase complementation) vector using the KpnI and SalI sites. The full-length CDSs of *MdPP2AA*, *MdPP2AAβ*, *MdPP2ABβ-2*, *MdPP2AB’β-2*, *MdPP2AB’γ-1*, *MdPP2AC-10*, and *MdSnRK2.6* without stop codons were fused to the pC1300-cLUC vector using the KpnI and SalI sites. The LUC signal was detected according to Chen and Wang (2022) [97].

### Western blotting

Total proteins of epidermal strips from apple leaves were extracted according to Chen and Wang (2022) [97]. Protein extractions (80 μg) containing 5× loading buffer were heated and denatured at 95°C, then separated by 10% SDS–PAGE. Western blotting was carried out according to Chen *et al*. (2014) [91]. The blots were probed with a customized anti-MdPP2AC peptide-raised (SHSDLDRQIEHLMEC) antibody to MdPP2AC (Abclonal Biotechnology Co., Ltd.) (Appendix 3) and a phosphorylated anti-PP2AC antibody (Abclonal Biotechnology Co., Ltd.; Catalog No. AP1043) (Appendix 4). When the MdPP2AC antibody was customized, its potential specificity was evaluated. Among the 10 MdPP2Acs, only the antigen sequences of MdPP2AC5 and MdPP2AC were the same, whereas the others were different; interference of the other 8 MdPP2Acs could therefore be excluded **(**Fig. S8A**)**. The possibility of interference between PP2AC and PP2AC5 is less likely, because RT-qPCR results **(**Fig. S8B and C) showed that the relative expression of the latter protein had no correlation with protein abundance **(**Fig. 3A and C). Therefore, the customized MdPP2AC antibody was able to discriminate the MdPP2AC protein. During the experiment, the dilution of the primary antibody was 1:1000, and that of the secondary antibody was 1:5000. Horseradish peroxidase (HRP) substrate solution (Merck Millipore, Cat. No. WBKLS0100) was used to detect chemiluminescence signals. Anti-α-tubulin antibody was obtained from immunized mice, and anti-MdPP2AC antibody was obtained from immunized rabbits. An anti-plant α-tubulin antibody (Yeasen Biotechnology (Shanghai) Co., Ltd., Cat. No. 30304ES40) was used as an internal reference. The experiment was independently repeated three times. A Bio-Rad ChemiDoc Image System (model no. 12003153) was used to capture images for analysis of the effect of ALA and ABA on MdPP2AC protein accumulation using ImageJ software.

Similarly, the phosphorylation of MdSnRK2.6 was detected by western blotting. The SnRK2.6 phosphorylated antibody was obtained from Shanghai Center for Plant Stress Biology and CAS Center of Excellence in Molecular Plant Sciences, courtesy of Zhao Yang’s team at the Chinese Academy of Sciences [50]. During the experiment, the dilution of the SnRK2.6 antibody was 1:2000, and that of the secondary antibody was 1:5000.

### Expression of *MdPP2AC* and *MdSnRK2.6*

The cDNA of *MdPP2AC* was cloned into pGEX-4 T-1 (Amersham Pharmacia Biotech) using the BamHI and XhoI sites for GST fusion. The MdSnRK2.6 cDNA fragment was PCR-amplified and inserted in-frame at the BamHI and HindIII sites of pET28a (Novagen) for fusion to a 6× His tag. Expression of *MdPP2AC* and *MdSnRK2.6* was performed according to Chen *et al*. (2014) [91].

### Pull-down assay

The Pull-down assay was performed as previously described [91] to further verify the interaction between MdPP2AC and MdSnRK2.6 *in vitro*. Samples were separated on 10% SDS–PAGE gels and analyzed by western blotting using an anti-GST antibody (Abclonal Biotechnology Co., Ltd. Catalog No. AE001; 1:1000, v/v) or anti-His antibody (Abclonal Biotechnology Co., Ltd. Catalog No. AE003; 1:1000, v/v).

### Microscale thermophoresis for MdPP2AC and MdSnRK2.6 affinity

Microscale thermophoresis (MST) analysis was used to measure the affinity between MdPP2AC and MdSnRK2.6. Purified GST-MdPP2AC protein was fluorescently labeled using the Monolith RED-NHS secondary protein tag kit (Nano Temper Technology Co., Ltd., Cat# MO-L011). The NHS ester carried by the RED dye can bind covalently to primary amines (lysine residues), which can then be detected by the red-light detector of the Monolith NT.115 instrument. The labeled fluorescent MdPP2AC as a target protein was dissolved in 0.05% PBS Tween buffer to a concentration of 100 nM. Increasing concentrations of the MdSnRK2.6 ligand protein (ranging from 3.81 × 10^−4^ to 25 nM) were sequentially added to the MdPP2AC solution. The resulting mixtures were loaded into capillaries and measured using the Monolith NT.115 instrument. Molecular Affinity Analysis software (version 2.3) was used to process the data and construct a curve that enabled calculation of the dissociation constant.

### Vector construction and transient transformation of apple leaves

*MdPP2AC* and *MdSnRK2.6* cDNA fragments with stop codons were amplified by PCR and cloned into PBI121 using the XbaI and BamHI sites for GUS fusion. Recombinant plasmids for the transient overexpression of PBI121-*MdPP2AC*-GUS and PBI121-*MdSnRK2.6*-GUS were obtained. *MdPP2AC* and *MdSnRK2.6* overexpression or silencing constructs were generated according to Chen and Wang (2022) [97].

### Identification of positive transiently transgenic apple leaves

The positive transiently transgenic apple leaves were identified according to Chen and Wang (2022) [97].

### Measurement of endogenous Ca^2+^, H_2_O_2_, and flavonols in guard cells

Intracellular Ca^2+^ and endogenous H_2_O_2_ levels in guard cells were measured according to An *et al*. (2016a, b) [19, 20]. Endogenous flavonols were measured according to Watkins *et al*. (2014) [102]. Fluorescence intensity was measured using an ultra-resolution laser confocal microscope (LSM 800, ZEISS, Germany) with the following parameters: excitation light 488 nm, emitted light 525 ± 15 nm, power 50%, zoom 16, mild scanning, and frame 512 × 512. Relative fluorescence intensity of Fluo-3 a.m., H_2_DCF-DA, and DPBA was measured with ImageJ software.

### Determination of endogenous ALA content in apple leaves

The test-tube apple plantlets were cultured on MS medium containing 20% PEG-6000 for 3 days in a growth chamber with temperature of 20–25°C, photoperiod of 16 h a day and PPFD of 240 μmol m^−2^ s^−1^. Then the leaf epidermis was collected to measure the ALA content according to Zhang (2011) [77].

### Statistics and reproducibility

The data are means of at least three independent biological replications. Statistical analyses were performed using a two-sided Student’s *t*-test or a one-way ANOVA followed by mean separation with Tukey’s honestly significant difference test or Duncan’s multiple range test.

## Acknowledgements

This work was supported by the Natural Science Foundation of China (32172512, 32272641), the Jiangsu Special Fund for Frontier Foundation Research of Carbon Peaking and Carbon Neutralization (BK20220005), the Jiangsu Agricultural Science and Technology Innovation Fund [CX(20)2023], and a project funded by the Priority Academic Program Development of Jiangsu Higher Education Institutions. The authors greatly appreciate Miss Anqi Xing and her professional editorial team A&L Scientific Editing (www.alpublish.com) for helping us to polish the manuscript. Thanks to Prof. Yang Zhao and Dr. Qingzhong Li of the Shanghai Center for Plant Stress Biology and CAS Center of Excellence in Molecular Plant Sciences, Chinese Academy of Sciences, for kindly providing AtSnRK2.6 phosphorylated antibody.

## Author contributions

LJW and YYA designed the experiments and revised the manuscript. ZC performed all the experiments and wrote the manuscript. All authors have read and approved the manuscript.

## Data availability

Data supporting the conclusions of this work are available in the paper and its supplementary materials. Some datasets were derived from sources in the public domain: the National Center for Biotechnology Information (https://www.ncbi.nlm.nih.gov) and the *Arabidopsis* information Resource (https://www.arabidopsis.org).

## Conflict of interest

None declared.

## Supplementary Data

Supplementary data is available at *Horticulture Research* online.

## Supplementary Material

Web_Material_uhad067
