# Supplementary material for: ALA reverses ABA-induced stomatal closure by modulating PP2AC and SnRK2.6 activity in apple leaves
Source: Hortic Res. 2023 Apr 10;10(6):uhad067. doi: 10.1093/hr/uhad067 (PMC10243991; doi:10.1093/hr/uhad067)
Supplement: Web_Material_uhad067 [file web_material_uhad067.zip › Appendix 1 PP2A activity kits.pdf]

Plant PP2A

FOR RESEARCH USE ONLY

Assay range： 5IU/L - 200IU/L **96 determinations**

Purpose

This kit allows for the determination of PP2A activity in Plant cell, tissue and other biological fluids.

Principle of the assay

The kit assay Plant PP2A level in the sample，use Purified Plant PP2A antibody to coat microtiter plate wells, make solid-phase antibody, then add PP2A to wells, Combined PP2A antibody which With HRP labeled, become antibody - antigen - enzyme-antibody complex, after washing Completely, Add TMB substrate solution,TMB substrate becomes blue color At HRP enzyme-catalyzed, reaction is terminated by the addition of a sulphuric acid solution and the color change is measured spectrophotometrically at a wavelength of 450 nm. The concentration of Plant PP2A in the samples is then determined by comparing the O.D. of the samples to the standard curve.

Materials provided with the kit

1	wash solution	20ml×1bottle	7	Stop Solution	6ml×1 bottle
2	HRP-Conjugate reagent	6ml×1 bottle	8	Standard （320IU/L）	0.5ml×1 bottle
3	Microelisa stripplate	12well×8strips	9	Standard diluent	1.5ml×1bottle
4	Sample diluent	6ml×1 bottle	10	Instruction	1
5	Chromogen Solution A	6ml×1 bottle	11	Closure plate membrane	2
6	Chromogen Solution B	6ml×1 bottle	12	Sealed bags	1

Specimen requirements

1. extract as soon as possible after Specimen collection,and according to the

http://www.affandi-e.com
订购热线：021-52961052

relevant literature, and should be experiment as soon as possible after the extraction. If it can't, specimen can be kept in -20 ℃ to preserve, Avoid repeated freeze-thaw cycles.

2. Can't detect the sample which contain NaN3, because NaN3 inhibits HRP active.

Assay procedure

1. Dilute and add sample:Dilute Original density Standard as follow table:

160IU/L	5 Standard	150μl Original density Standard+150μl Standard diluent
80IU/L	4 Standard	150μl 5 Standard+150μl Standard diluent
40IU/L	3 Standard	150μl 4 Standard+150μl Standard diluent
20IU/L	2 Standard	150μl 3 Standard +150μl Standard diluent
10IU/L	1 Standard	150μl 2 Standard +150μl Standard diluent

2.add sample： Set blank wells separately (blank comparison wells don't add sample and HRP-Conjugate reagent, other each step operation is same). testing sample well. Add 50µl of standard to Microelisa stripplate, add Sample dilution 40µl to testing sample well, then add testing sample 10µl (sample final dilution is 5-fold), add sample to wells , don't touch the well wall as far as possible, and Gently mix.

3.Incubate: After closing plate with Closure plate membrane ,incubate for 30 min at 37℃.

4.Configurate liquid: 30-fold(or 20-fold) wash solution diluted 30-fold (or 20-fold) with distilled water and reserve.

5.washing： Uncover Closure plate membrane, discard Liquid, dry by swing, add washing buffer to every well, still for 30s then drain, repeat 5 times, dry by pat.

6.add enzyme：Add HRP-Conjugate reagent 50µl to each well, except blank well.

7.incubate：Operation with 3.

8.washing：Operation with 5.

订购热线：021-52961052
传真：021-57690166
http://www.affandi-e.com

9.color：Add Chromogen Solution A 50ul and Chromogen Solution B 50ul to each well, evade the light preservation for 10 min at 37℃

10.Stop the reaction：Add Stop Solution50μl to each well, Stop the reaction(the blue color change to yellow color).

11.assay：take blank well as zero , Read absorbance at 450nm after Adding Stop Solution and within 15min.

Steps description

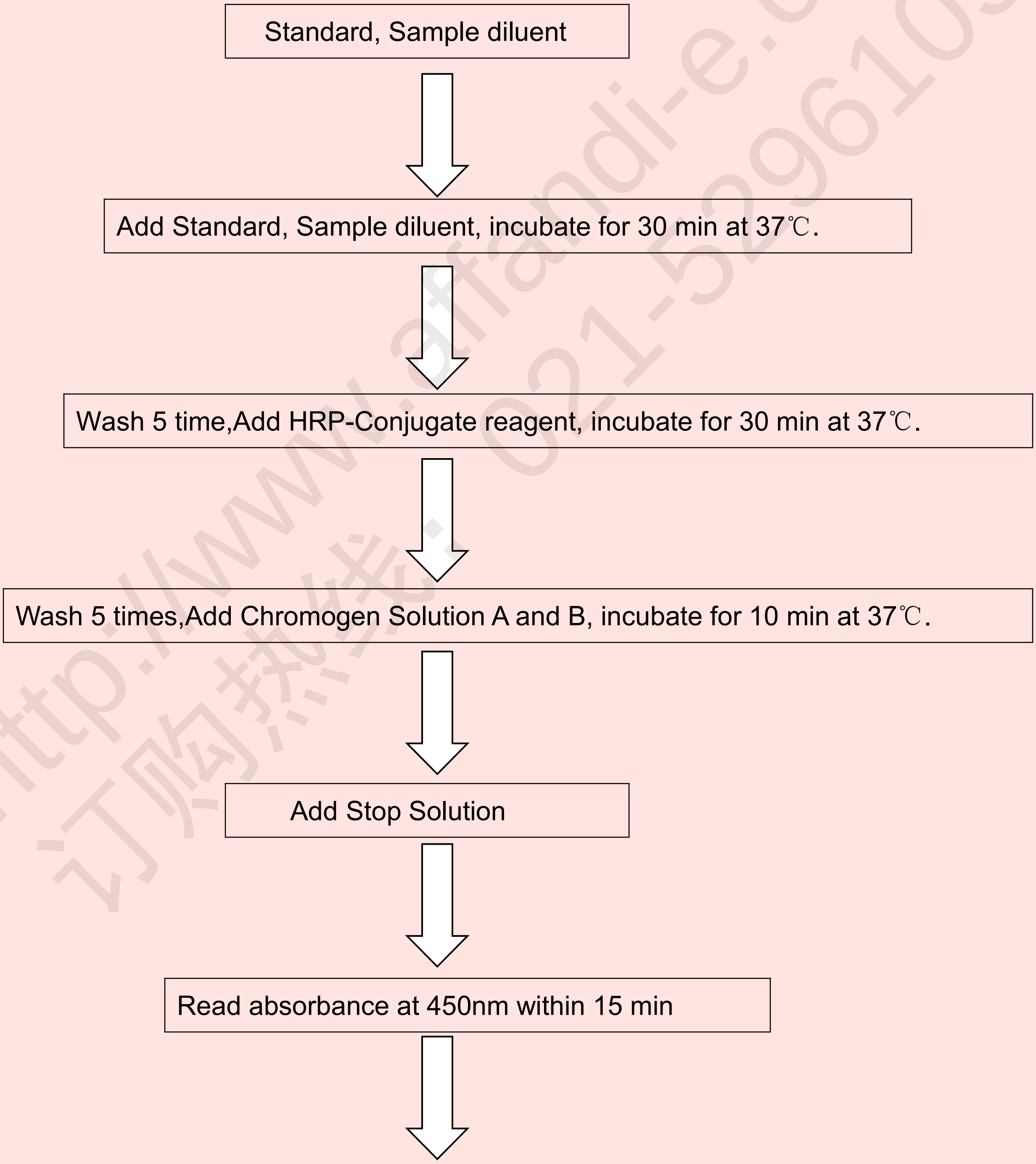

http://www.affandi-e.com 订购热线：021-52961052

calculate

Calculate

Take the standard density as the horizontal, the OD value for the vertical ,draw the standard curve on graph paper, Find out the corresponding density according to the sample OD value by the Sample curve, multiplied by the dilution multiple, or calculate the straight line regression equation of the standard curve with the standard density and the OD value ,with the sample OD value in the equation, calculate the sample density, multiplied by the dilution factor, the result is the sample actual density.

Important notes

1. The kit takes out from the refrigeration environment should be balanced 15-30 minutes in the room temperature, ELISA plates coated if has not use up after opened, the plate should be stored in Sealed bag.
2. washing buffer will Crystallization separation, it can be heated the water helps dissolve when dilute . Washing does not affect the result.
3. add Sample with sampler Each step, And proofread its accuracy frequently, avoids the experimental error. add sample within 5 min, if the number of sample is much , recommend to use Volley .
4. if the testing material content is excessively higher (The sample OD is bigger than the first standard well),please dilute Sample (n-fold), Please diluente and multiplied by the dilution factor.（×n×5）.
5. Closure plate membrane only limits the disposable use, to avoid cross-contamination.
6. The substrate evade the light preservation.
7. Please according to use instruction strictly, The test result determination must take the microtiter plate reader as a standard.
8. All samples, washing buffer and each kind of reject should according to

http://www.affandi-e.com
订购热线：021-52961052

infective material process.

9. Do not mix reagents with those from other lots.

Storage and validity

1. Storage： 2-8℃.
2. validity： six months

http://www.affandi-e.com
订购热线：021-52961052
