# Supplementary material for: ALA reverses ABA-induced stomatal closure by modulating PP2AC and SnRK2.6 activity in apple leaves
Source: Hortic Res. 2023 Apr 10;10(6):uhad067. doi: 10.1093/hr/uhad067 (PMC10243991; doi:10.1093/hr/uhad067)
Supplement: Web_Material_uhad067 [file web_material_uhad067.zip › Appendix 3 Customized MdPP2AC antibody.pdf]

Antigen Analysis and Design Report

1. Client Information

Client Information	
Customer Name: Zheng Chen	Tel:18852088927
Email:1449407079@qq.com	principal investigator:Liangju Wang
Institution/Department: Nanjing Agricultural University	Address:

2. Target Information

Basic Protein Information	
Gene Name: PP2A	Species: *Malus domestica*
GeneID:	Uniport ID: A0A498I7B3
NCBI NM NO:	NCBI NP NO:
Gene Size (bp):921 bp	Protein length (aa):306 aa
**Protein Background**	
No	

3. Antigen Design

3.1 Sequence Information

Nucleotide Sequence

>PP2A
ATGCCGTCTCATTCGGATCTGGACCGTCAGATCGAGCATCTGATGGAGTGCAAGACGTTGCCGGAGGCGGAGGTGA
AGACGCTGTGCGAGCAGGCGAGGGCGATCCTGGTGGAGGAGTGGAACGTGCAGCCGGTGAAGTGCCCCGTCACGGT
GTGCGGAGATATACACGGCCAGTTCTACGACCTCATTGAGCTTTTTAGGATAGGAGGGAACGCTCCCGATACTAAT
TACCTTTTTATGGGTGATTATGTAGATCGTGGGTACTATTCTGTGGAGACTGTCACGCTTCTGGTCGCTCTGAAAG
TCCGTTATAGAGATAGAATTACAATCCTCAGAGGAAATCACGAAAGCCGGCAAATTACTCAAGTGTATGGTTTTTA
TGACGAATGCTTGAGAAAGTATGGGAATGCCAATGTCTGGAAGTTCTTTACCGATTTATTTGATTATCTTCCCCTG
ACAGCCCTCATTGAGAGTCAGGTCTTCTGTTTGCATGGGGGTCTTTCACCATCTTTGGACACATTGGACAATATCC
GAGCTTTGGACCGTATACAGGAGGTTCCACATGAAGGACCAATGTGTGATCTCTTGTGGTCTGATCCAGATGACCG
CTGTGGGTGGGGAATATCTCCGCGGGGTGCTGGCTATACATTCGGGCAGGATATAGCTGCTCAGTTTAACCATACC
AATGGACTGAGTCTGATTTCAAGAGCTCATCAGCTTGTCATGGAAGGATACAATTGGTGCCAGGAAAAGAATGTGG
TGACCGTTTTCAGCGCTCCAAACTATTGCTATCGGTGTGGGAACATGGCCGCAATTTTGGAAATTGGCGAGAACAT
GGACCAGAATTTTCTGCAGTTCGACCCAGCCCCTCGTCAAATTGAGCCGGACAACACACGCAAGACTCCCGATTAT
TTTTTGTAA

Amino Acid Sequence
>PP2A<br>MPSHSDLDRQIEHLMECKTLPEAEVKTLCEQARAILVEEWNVQPVKCPVTVCGDIHGQFYDLIELFRIGGNAPDTN<br>YLFMGDYVDRGYYSVETVTLLVALKVRYRDRITILRGNHESRQITQVYGFYDECLRKYGNANVWKFFTDLFDYLPL<br>TALIESQVFCLHGGLSPSLDTLDNIRALDRIQEVPHEGPMCDLLWSDPDDRCGWGISPRGAGYTFGQDIAAQFNHT<br>NGLSLISRAHQLVMEGYNWCQEKNVVTVFSAPNYCYRCGNMAAILEIGENMDQNFLQFDPAPRQIEPDNTRKTPDY<br>FL

3.2 Sequence Specificity and Conservation

Protein Sequence Specificity

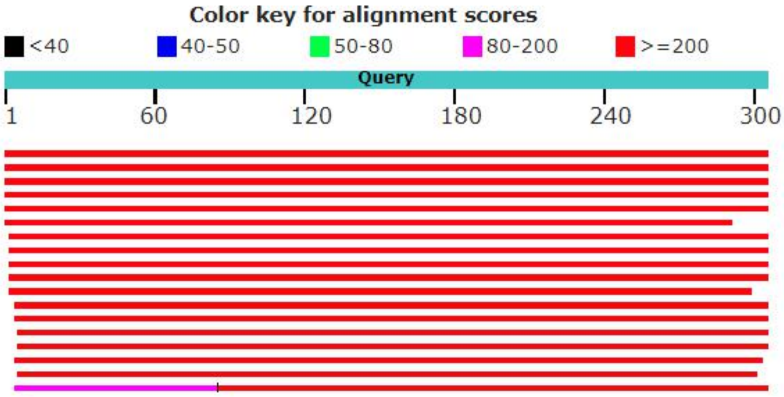

The PP2A amino acid sequence was analyzed within *Malus domestica* to determine whether antibodies produced against this protein are likely to recognize other proteins. An alignment of the PP2A sequence in NCBI shows that the sequence identity with another protein is high. Alignment results are shown below:

serine/threonine-protein phosphatase PP2A-4 catalytic subunit [Malus domestica]

Sequence ID: XP_008370945.1 Length: 313 Number of Matches: 1

▶ See 1 more title(s)

Range 1: 10 to 313 GenPept Graphics ▼ Next Match ▲ Previous Match

Score	Expect	Method	Identities	Positives	Gaps
522 bits(1344)	0.0	Compositional matrix adjust.	239/304(79%)	270/304(88%)	0/304(0%)

```
Query  3    SHSDLDRQIEHLMECKTLPEAEVKTLCEQARAILVEEWNVQPVKCPVTVCGDIHGQFYDL  62
            ++SDL+ QIE LM+CK L E +V+ LCE+A+ IL+ E NVQPVK PVT+CGDIHGQF+DL
Sbjct  10   TNSDLNEQIEQLMQCKPLSEQQVRMLCEKAKEILMGESNVQPVKSPVTICGDIHGQFHDL  69

Query  63   IELFRIGGNAPDTNYLFMGDYVDRGYYSVETVTLLVALKVRYRDRITILRGNHESRQITQ  122
             ELFRIGG  PDTNYLFMGDYVDRGYYSVETVTLLVALKVRY  RITILRGNHESRQITQ
Sbjct  70   AELFRIGGKCPDTNYLFMGDYVDRGYYSVETVTLLVALKVRYPQRITILRGNHESRQITQ  129

Query  123  VYGFYDECLRKYGNANVWKFFTDLFDYLPLTALIESQVFCLHGGLSPSLDTLDNIRALDR  182
            VYGFYDECLRKYGNANVWK FTDLFDY PLTAL+ES++FCLHGGLSPS++ LDNIR  DR
Sbjct  130  VYGFYDECLRKYGNANVWKIFTDLFDYFPLTALVESEIFCLHGGLSPSIENLDNIRNFDR  189

Query  183  IQEVPHEGPMCDLLWSDPDDRCGWGISPRGAGYTFGQDIAAQFNHTNGLSLISRAHQLVM  242
            +QEVPHEGPMCDLLWSDPDDRCGWGISPRGAGYTFGQDI+ QFNHTN L LI+RAHQLVM
Sbjct  190  VQEVPHEGPMCDLLWSDPDDRCGWGISPRGAGYTFGQDISEQFNHTNNLKLIARAHQLVM  249

Query  243  EGYNWCQEKNVVTVFSAPNYCYRCGNMAAILEIGENMDQNFLQFDPAPRQIEPDNTRKTP  302
            +G+NW  E+ VVT+FSAPNYCYRCGNMA++LE+ +      F+QF+PAPR+ EPD TR+TP
Sbjct  250  DGFNWAHEQKVVTIFSAPNYCYRCGNMASVLEVDDCRGHTFIQFEPAPRRGEPDVTRRTP  309

Query  303  DYFL  306
            DYFL
Sbjct  310  DYFL  313
```

Sequence Conservation
NO

3.3 Protein Expressivity Analysis

Transmembrane Domain Prediction

```
# PP2A Length: 306
# PP2A Number of predicted TMHs:  0
# PP2A Exp number of AAs in TMHs: 0.07345
# PP2A Exp number, first 60 AAs:  0
# PP2A Total prob of N-in:        0.02352
PP2A    TMHMM2.0        outside      1   306
```

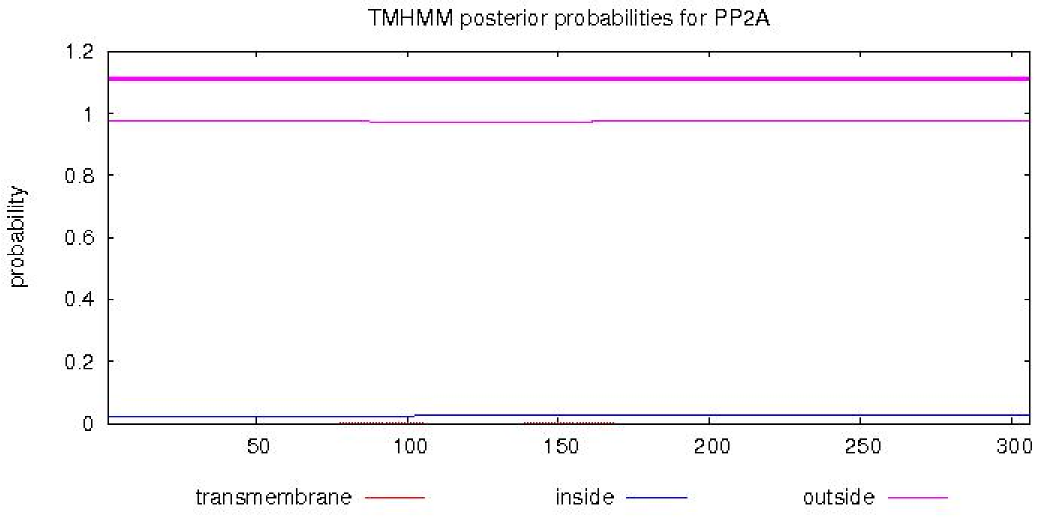

Note: Transmembrane domains/structures should normally be avoided when designing antigens.

Posttranslational Modifications (PTMs)

NO

Note: As signal peptides often exhibit strong hydrophobicity and may limit protein expression, they should normally be excluded from antigens.

Conserved Domains

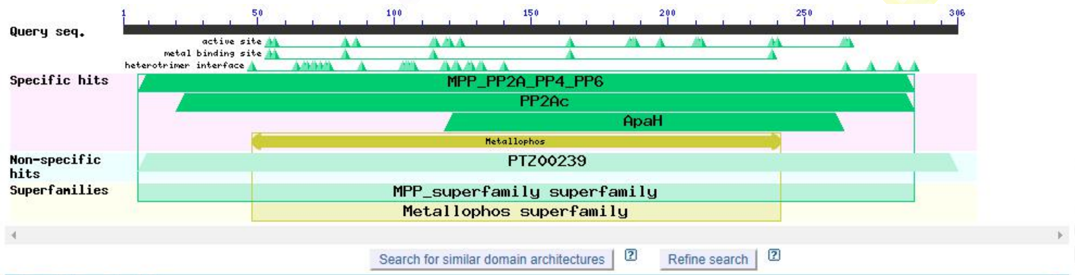

List of domain hits

Name	Accession	Description	Interval	E-value
[+] MPP_PP2A_PP4_PP6	cd07415	PP2A, PP4, and PP6 phosphoprotein phosphatases, metallophosphatase domain; PP2A-like family of ...	6-290	0e+00
[+] PTZ00239	PTZ00239	serine/threonine protein phosphatase 2A; Provisional	6-306	4.37e-155
[+] PP2Ac	smart00156	Protein phosphatase 2A homologues, catalytic domain; Large family of serine/threonine ...	20-290	3.55e-140
[+] ApaH	COG0639	Diadenosine tetraphosphatase ApaH/serine/threonine protein phosphatase, PP2A family [Signal ...	118-264	7.06e-41
[+] Metallophos	pfam00149	Calcineurin-like phosphoesterase; This family includes a diverse range of phosphoesterases, ...	48-241	3.13e-32

Note: Normally, antigens should incorporate whole domains.

3D Structure Analysis

NO

Note: 3D structure is helpful for identification of B-Cell epitopes and for analyzing protein expressibility.

Secondary Structure Prediction

```
                    10         20         30         40         50         60         70         80         90        100
                     |          |          |          |          |          |          |          |          |          |
UNK_256170 MPSHSDLDRQIEHLMECKTLPEAEVKTLCEQARAILVEEWNVQPVKCPVTVCGDIHGQFYDLIELFRIGGNAPDTNYLFMGDYVDRGYYSVETVTLLVAL
DSC        cccccchhhhhhhhhccchhhhhhhhhhhhhhhhhhhhcccccccccccccccccccchhhhhhhhhhcccccccceeecccccccccchhhhhhhhhhh
MLRC       cccchhhhhhhhhhhhccccchhhhhhhhhhhhhhhhhcccccccccceeecccccchhhhhhhhhhcccccccccceeeecccccccccchhhhhhhhhh
PHD        ????????????????????????????????????????????????????????????????????????????????????????????????????
Sec.Cons.  cccc??hhhhhhhhh?cc???hhhhhhhhhhhhhhhhhcccccccccc???cccccc?hhhhhhhhh?cccccccceee?ccccccccc?hhhhhhhhhh

                   110        120        130        140        150        160        170        180        190        200
                     |          |          |          |          |          |          |          |          |          |
UNK_256170 KVRYRDRITILRGNHESRQITQVYGFYDECLRKYGNANVWKFFTDLFDYLPLTALIESQVFCLHGGLSPSLDTLDNIRALDRIQEVPHEGPMCDLLWSDP
DSC        hcccccceeecccccccccccccccchhhhhhccccchhhhhhhhhhcchhhccccceeeecccccccccccccchhhhhhhccccccccccccccccc
MLRC       hccccceeeeeecccccchhhhhhhhhhhhhhhccccchhhhhhhhhhhhhhhhhhhhheeeecccccccahhhhhhhhhhhhhcccccccceeeeeeccc
PHD        ????????????????????????????????????????????????????????????????????????????????????????????????????
Sec.Cons.  hccccc?eee??cccccc?????????hhhhhhccccchhhhhhhhhh??hhh??????eee?ccccccc??????hhhhhhhcccccccc??????ccc

                   210        220        230        240        250        260        270        280        290        300
                     |          |          |          |          |          |          |          |          |          |
UNK_256170 DDRCGWGISPRGAGYTFGQDIAAQFNHTNGLSLISRAHQLVMEGYNWCQEKNVVTVFSAPNYCYRCGNMAAILEIGENMDQNFLQFDPAPRQIEPDNTRK
DSC        ccccccccccccccccccchhhhhhhhccchhhhhhhhhhhhhhhhhccccceeeecccccccccccchhhhhhhhhccccccccccccccccccccccc
MLRC       cccccccccccccccccchhhhhhhcccccchhhhhhhhhhhhccccccccceeeeeecccccccccchheeeehchccccceeeecccccccccccccc
PHD        ????????????????????????????????????????????????????????????????????????????????????????????????????
Sec.Cons.  cccccccccccccccccc?hhhhhh??ccc?hhhhhhhhhhhh????ccccceeee??cccccccccchh????h?hccccc????cccccccccccccc

UNK_256170 TPDYFL
DSC        cccccc
MLRC       cccccc
PHD        ??????
Sec.Cons.  cccccc
```

Note: e: Extended strand; c: Random coil; h: Alpha helix.

Note: Secondary structure is helpful for identification of B-Cell epitopes and for analyzing protein expressibility.

3.4 Protein Antigenicity Analysis

Prediction of Linear B-Cell Epitopes

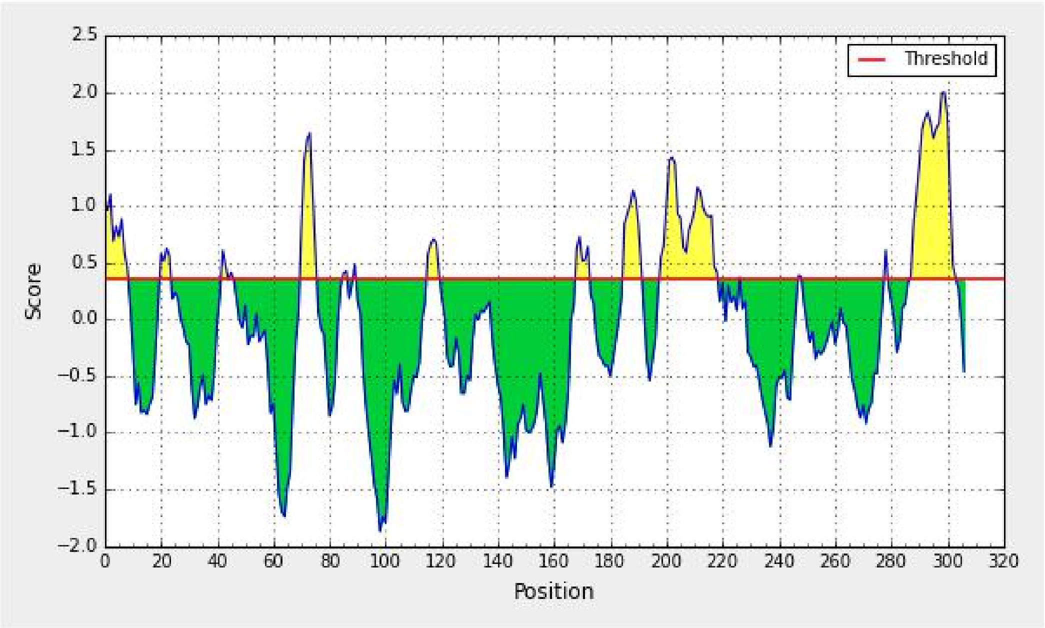

Note: Prediction of linear B-Cell epitopes is helpful to understand protein hydrophobicity.

3.5 Antigen Suggestions

Antigen Route	Recommend Region	Basis for Selection
Protein fragment	No	The target protein sequence have high homology with serine/threonine-protein phosphatase PP2A-4 catalytic subunit protein.
Synthetic Peptide	2-17 aa	(1) Antigen peptide and purification peptide:SHSDLDRQIEHLMEC<br>(2) Screening peptide and purification peptide: SHGNLDEQIAQLMQC

4. Client Requirements

Selected clonality for this antibody is: rabbit/mouse polyclonal/monoclonal antibody.

Note: This report and all statements within it are confidential. Distribution and disbursement without prior permission from ABclonal is strictly prohibited.
