# Supplementary material for: ALA reverses ABA-induced stomatal closure by modulating PP2AC and SnRK2.6 activity in apple leaves
Source: Hortic Res. 2023 Apr 10;10(6):uhad067. doi: 10.1093/hr/uhad067 (PMC10243991; doi:10.1093/hr/uhad067)
Supplement: Web_Material_uhad067 [file web_material_uhad067.zip › Appendix 4 Phosphorylated antibody.pdf]

Leader in Biomolecular Solutions for Life Science

www.abclonal.com

Phospho-PP2A alpha-Y307 Rabbit mAb

Catalog No.: AP1043 **Recombinant**

Basic Information

Catalog No.
AP1043

Observed MW
35KDa

Calculated MW
35kDa

Category
Primary antibody

Applications
WB

Cross-Reactivity
Human, Mouse

Background

This gene encodes the phosphatase 2A catalytic subunit. Protein phosphatase 2A is one of the four major Ser/Thr phosphatases, and it is implicated in the negative control of cell growth and division. It consists of a common heteromeric core enzyme, which is composed of a catalytic subunit and a constant regulatory subunit, that associates with a variety of regulatory subunits. This gene encodes an alpha isoform of the catalytic subunit. [provided by RefSeq, Jul 2008]

Recommended Dilutions

**WB**	1:500 - 1:2000

Immunogen Information

Gene ID
5515

Swiss Prot
P67775

Immunogen
A phospho specific peptide corresponding to residues surrounding Y307 of human PP2A alpha

Synonyms
PP2Ac; PP2CA; PP2Calpha; RP-C

Contact

400-999-6126

cn.market@abclonal.com.cn

www.abclonal.com.cn

Product Information

Source
Rabbit

Isotype
IgG

Purification
Affinity purification

Storage
Store at -20℃. Avoid freeze / thaw cycles.
Buffer: PBS with 0.02% sodium azide,0.05% BSA,50% glycerol,pH7.3.

Validation Data

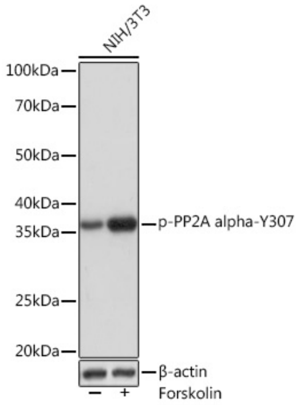

Western blot analysis of extracts of NIH/3T3 cells, using Phospho-PP2A alpha-Y307 Rabbit mAb (AP1043) at 1:1000 dilution.NIH/3T3 cells were treated by Forskolin (30 uM) at 37℃ for 30 minutes after serum-starvation overnight.
Secondary antibody: HRP Goat Anti-Rabbit IgG (H+L) (AS014) at 1:10000 dilution.
Lysates/proteins: 25ug per lane.
Blocking buffer: 3% BSA.
Detection: ECL Basic Kit (RM00020).
Exposure time: 3s.

For research use only. Not for therapeutic or diagnostic purposes.
Please visit http://abclonal.com for a complete listing of recommended products.
