# Supplementary material for: ALA reverses ABA-induced stomatal closure by modulating PP2AC and SnRK2.6 activity in apple leaves
Source: Hortic Res. 2023 Apr 10;10(6):uhad067. doi: 10.1093/hr/uhad067 (PMC10243991; doi:10.1093/hr/uhad067)
Supplement: Web_Material_uhad067 [file web_material_uhad067.zip › Supplementary figures--.docx]

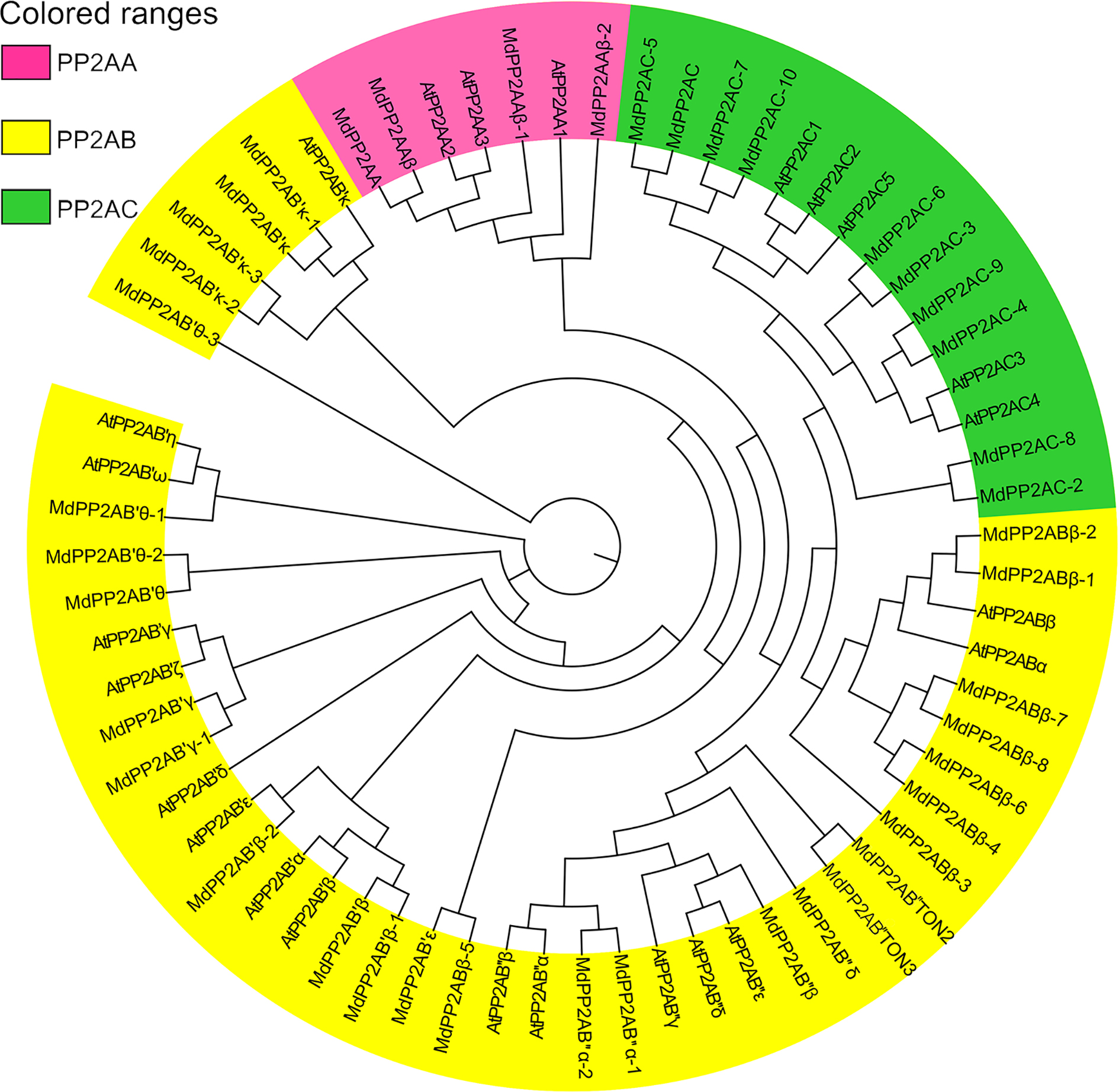


**Fig. S1. Unrooted phylogenetic tree of PP2A proteins from apple and *Arabidopsis*** constructed with the neighbor-joining method in MEGA 7.0 with 1000 bootstrap replicates. Three PP2A subfamilies are indicated by different colors.


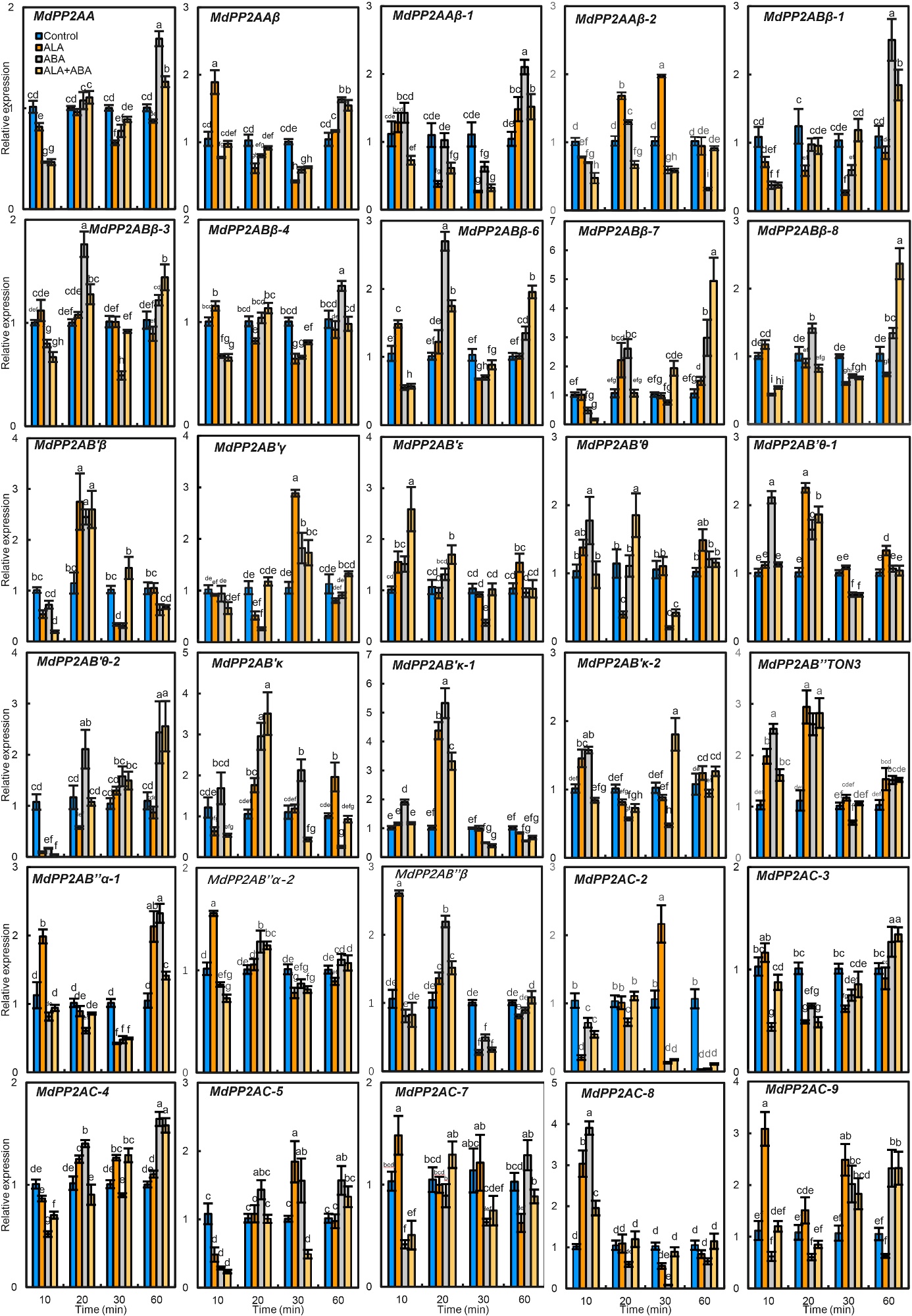
**Fig. S2. Expression analysis of *PP2A* genes encoding different subunits besides those listed in Fig. 2 in apple leaves after ALA, ABA, and ALA + ABA treatment.** Preparation and treatment of epidermal strips were described in Fig. 1, and data are the means of three independent biological replicates.


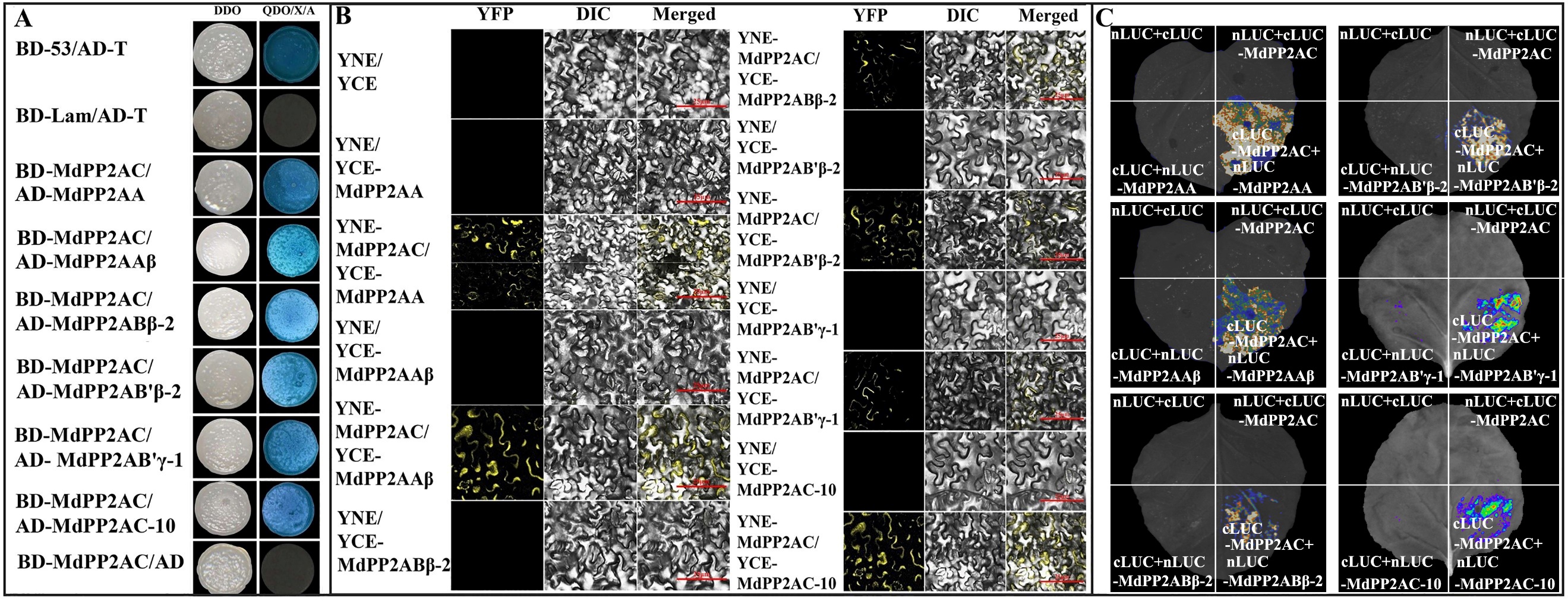


**Fig. S3** **Interactions of MdPP2AC with other MdPP2A subunits.** **A:** Yeast two-hybrid (Y2H) assays showed that MdPP2AC interacted with MdPP2AA, MdPP2AAβ, MdPP2ABβ-2, MdPP2AB'β-2, MdPP2AB'γ-1, and MdPP2AC-10. The combination of BD-53 plus AD-T was used as a positive control, and BD-Lam plus AD-T was used as a negative control, where BD was the pGBKT7 vector, AD was the pGADT7 vector, DDO was double dropout SD medium (SD/–Leu/–Trp), and QDO/X/A was quadruple dropout medium SD/–Ade/–His/–Leu/–Trp supplemented with X-a-Gal and AbA. **B:** Bimolecular fluorescence complementation analysis (BiFC) in tobacco confirmed that MdPP2AC interacted with the six other subunits identified in the Y2H assay. YCE is the C-terminal fragment of yellow fluorescent protein, and YNE is the N-terminal fragment. Co-expression of the YNE vector and the YCE vector with no insert/YCE empty vector with prey were used as negative controls. The yellow fluorescence signals were captured using a super resolution laser confocal microscope (LSM 800, ZEISS, Germany). Scale bar: 25 µm. **C:** Firefly luciferase complementation (FLC) assay. The recombinant plasmids were expressed in 4-week-old *N. benthamiana* leaves for 60 h, and the luciferase signals were captured using an *in vivo* imager (PIXIS 1024B, Princeton, USA). The combinations of nLUC plus cLUC, nLUC plus cLUC recombinant plasmid, and nLUC recombinant plasmid plus cLUC were used as negative controls. Three independent experiments were performed with similar results.


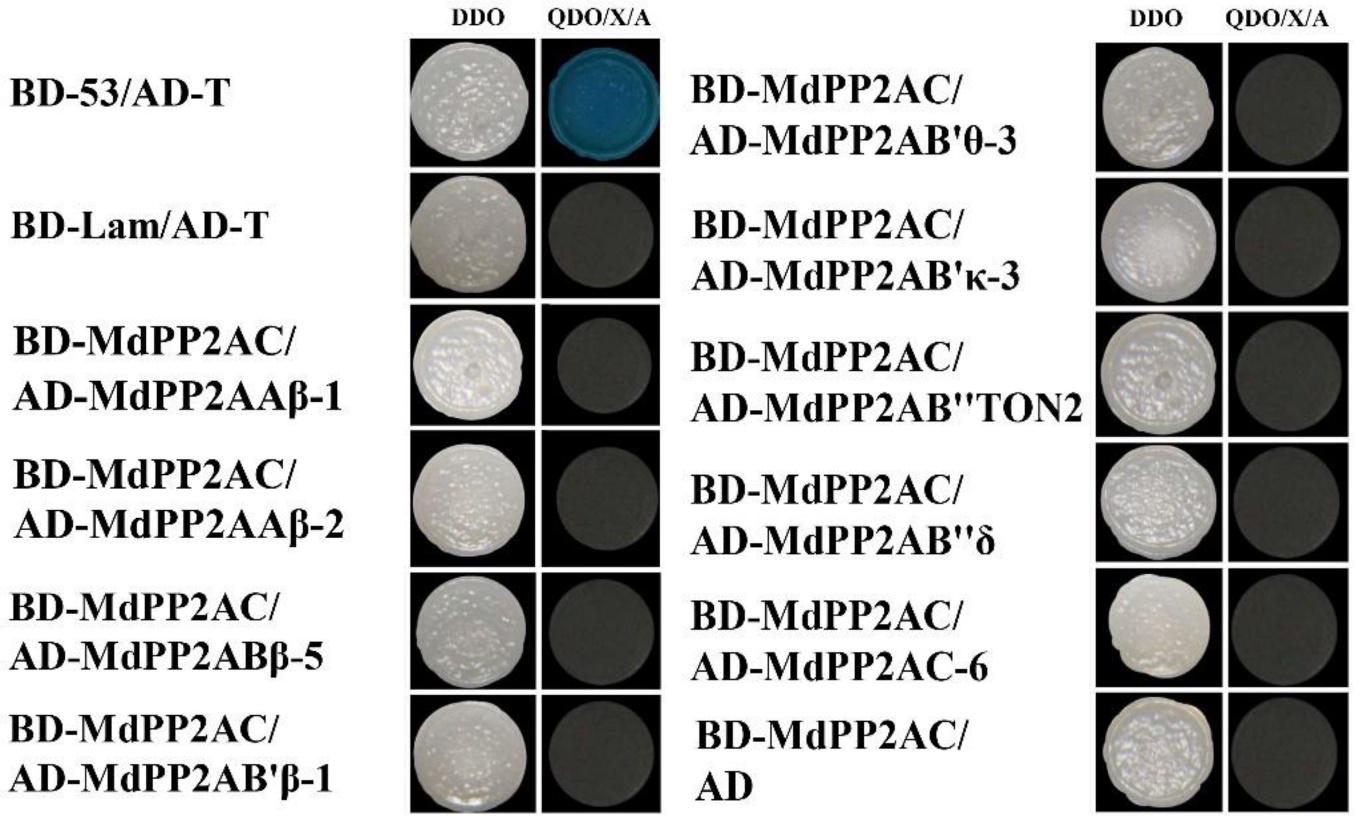


**Fig. S4.** **No interactions of MdPP2AC with other subunits.** The combination of BD-53 plus AD-T was used as a positive control, and BD-Lam plus AD-T was used as a negative control, where BD is the pGBKT7 vector, AD is the pGADT7 vector, DDO is double dropout SD medium (SD/–Leu/–Trp), and QDO/X/A is quadruple dropout medium SD/–Ade/–His/–Leu/–Trp supplemented with X-a-Gal and AbA.


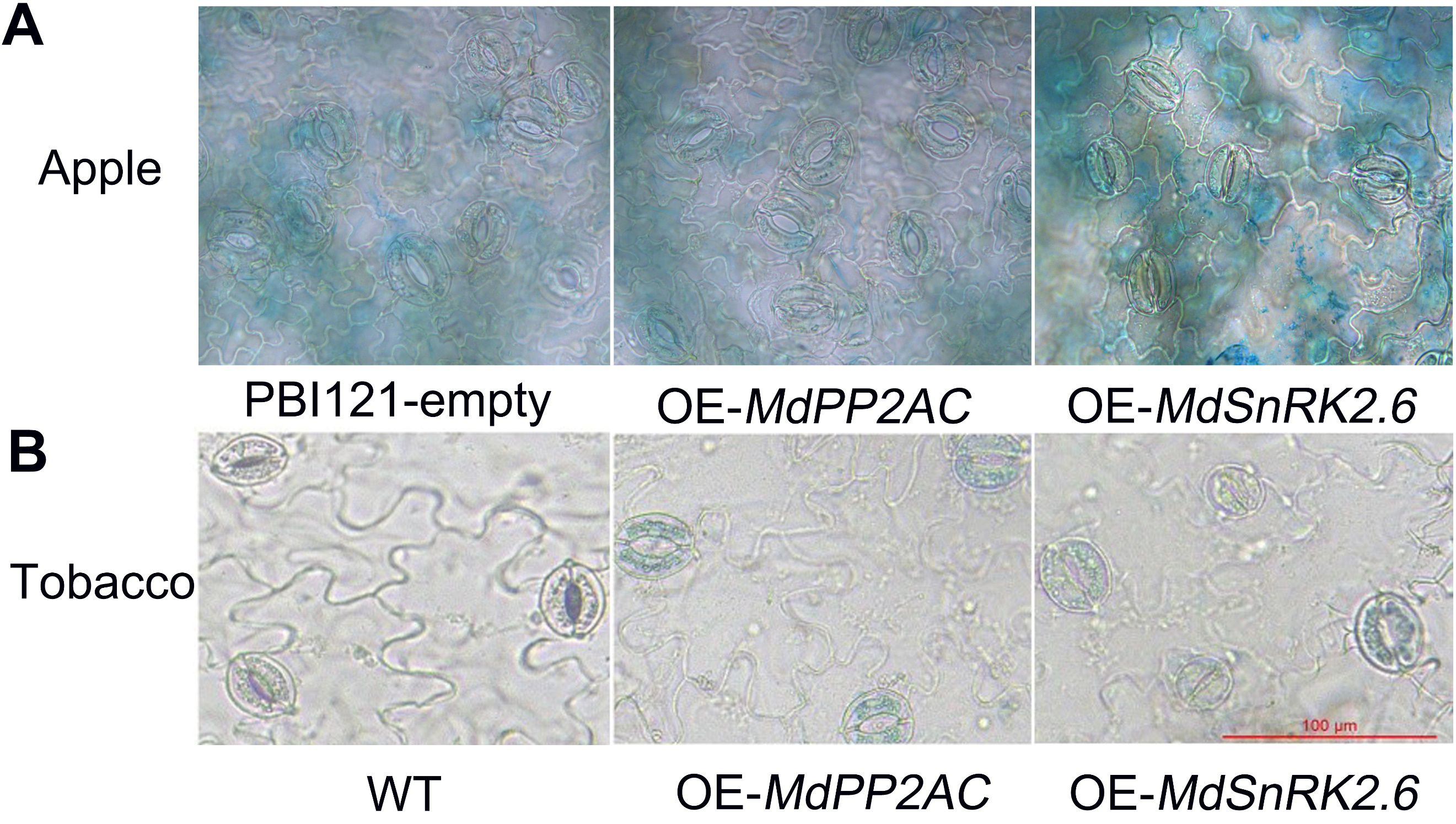


**Fig. S5. GUS detection of *MdPP2AC* and *MdSnRK2.6* expression in transiently transgenic apple leaves (A) and stable genetically modified tobacco leaves (B).** The blue staining in the guard cells of epidermis shows *MdPP2AC* and *MdSnRK2.6*::*GUS* expression in the transgenic leaves. Scale bar = 100 µm.


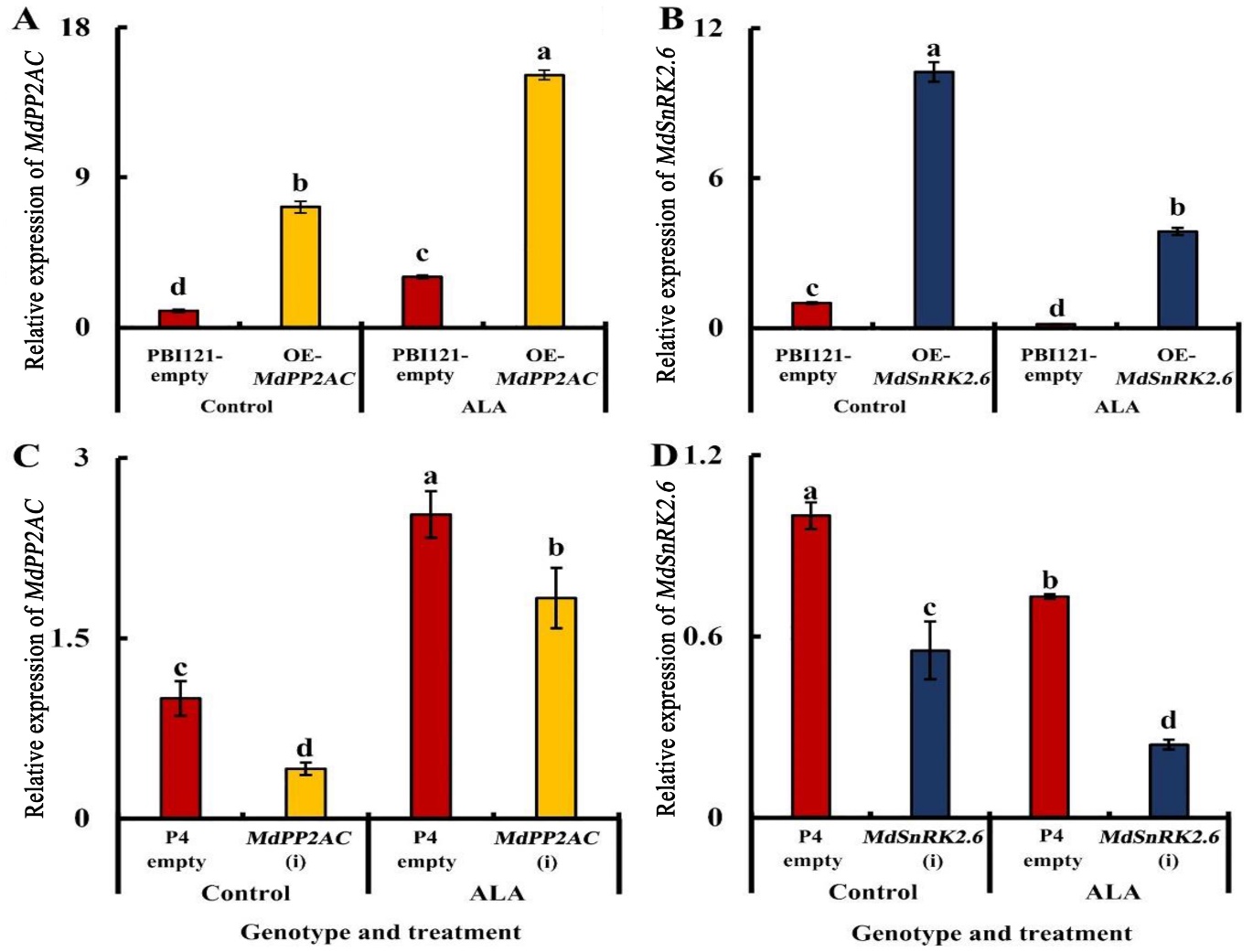


**Fig. S6. RT-qPCR analysis of *MdPP2AC* and *MdSnRK2.6* expressions in transiently transgenic apple leaves with or without exogenous ALA treatment.** **A** and **C**: Relative expression of *MdPP2AC* in OE-*MdPP2AC* and MdPP2AC(i). **B** and **D**: Relative expression of *MdSnRK2.6* in OE-*MdSnRK2.6* and MdSnRK2.6(i).


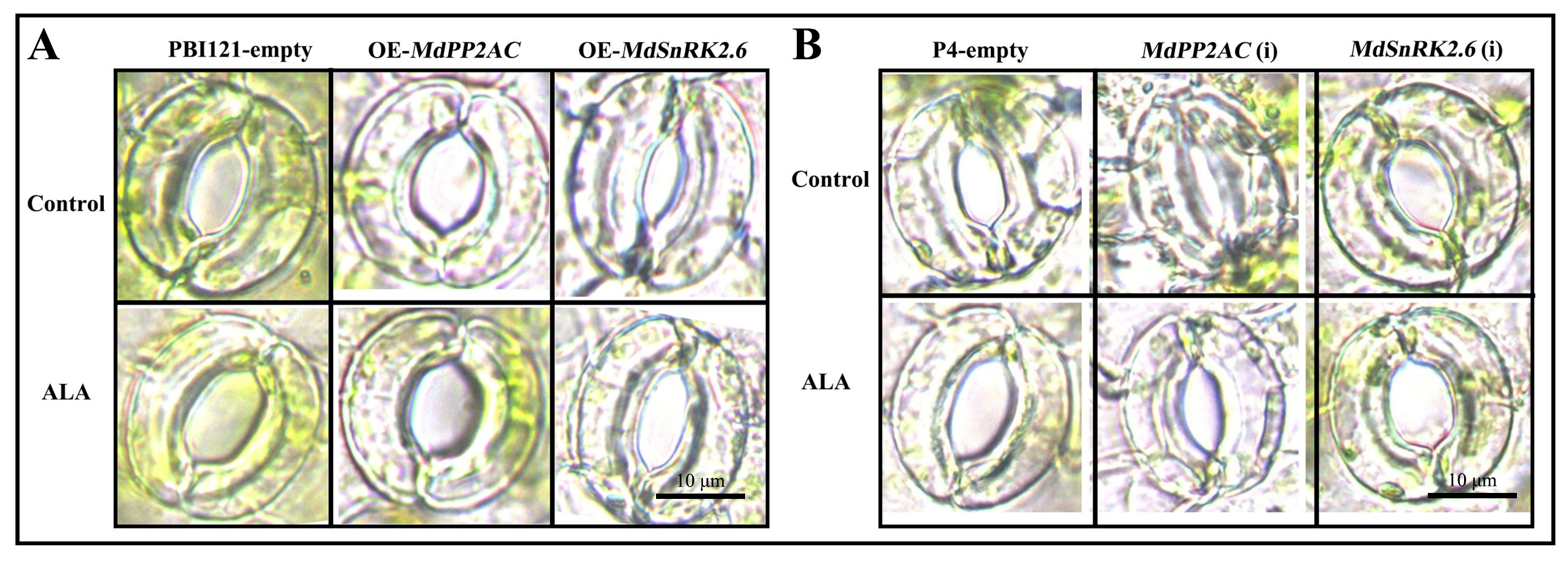


**Fig. S7 ALA treatment affects stomatal aperture in transiently transgenic apple leaves. A:** transiently transgenic apple leaves in which MdPP2AC or MdSnRK2.6 was overexpressed, treated with or without ALA; **B**: transiently transgenic apple leaves in which MdPP2AC or MdSnRK2.6 was partially silenced, treated with or without ALA. Scale bar = 10 µm. In all experiments, isolated epidermal strips were incubated at 25 °C in MES-KCl buffer without plant hormones (Control). After a 2-h illumination pretreatment (240 µmol m^−2^ s^−1^), the strips were transferred to the same MES-KCl buffer with 0.5 mg L^−1^ ALA and illuminated for 1 hr for taking stomatal photos of apple.


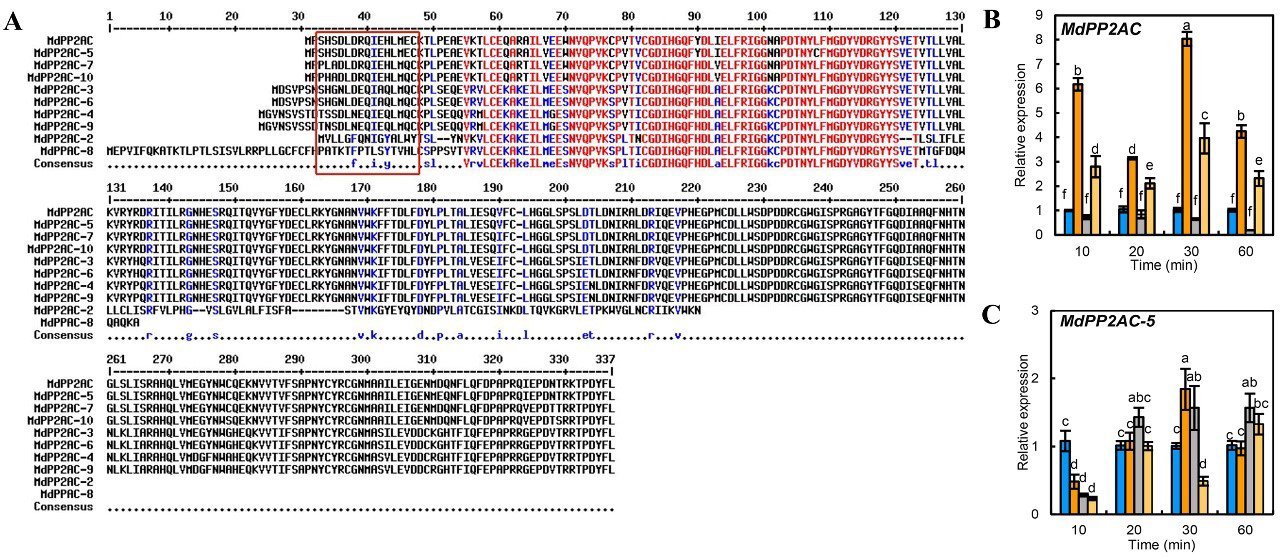


**Fig. S8. The customized antibody MdPP2AC specifically recognizes the protein MdPP2AC.** **A**: Comparison of the immunogen sequences of MPP2AC with those of nine other catalytic subunits. The amino acid sequence framed in the red box is the immunogen sequence. **B** and **C**: Expression analysis of MdPP2AC and MdPP2AC-5 in apple leaves after ALA, ABA, and ALA + ABA treatment. The epidermal strip preparation and treatments were described in Fig. 1, and data are the means of three independent biological replicates. The means sharing lowercase letters are not significantly different at *p* = 0.05.


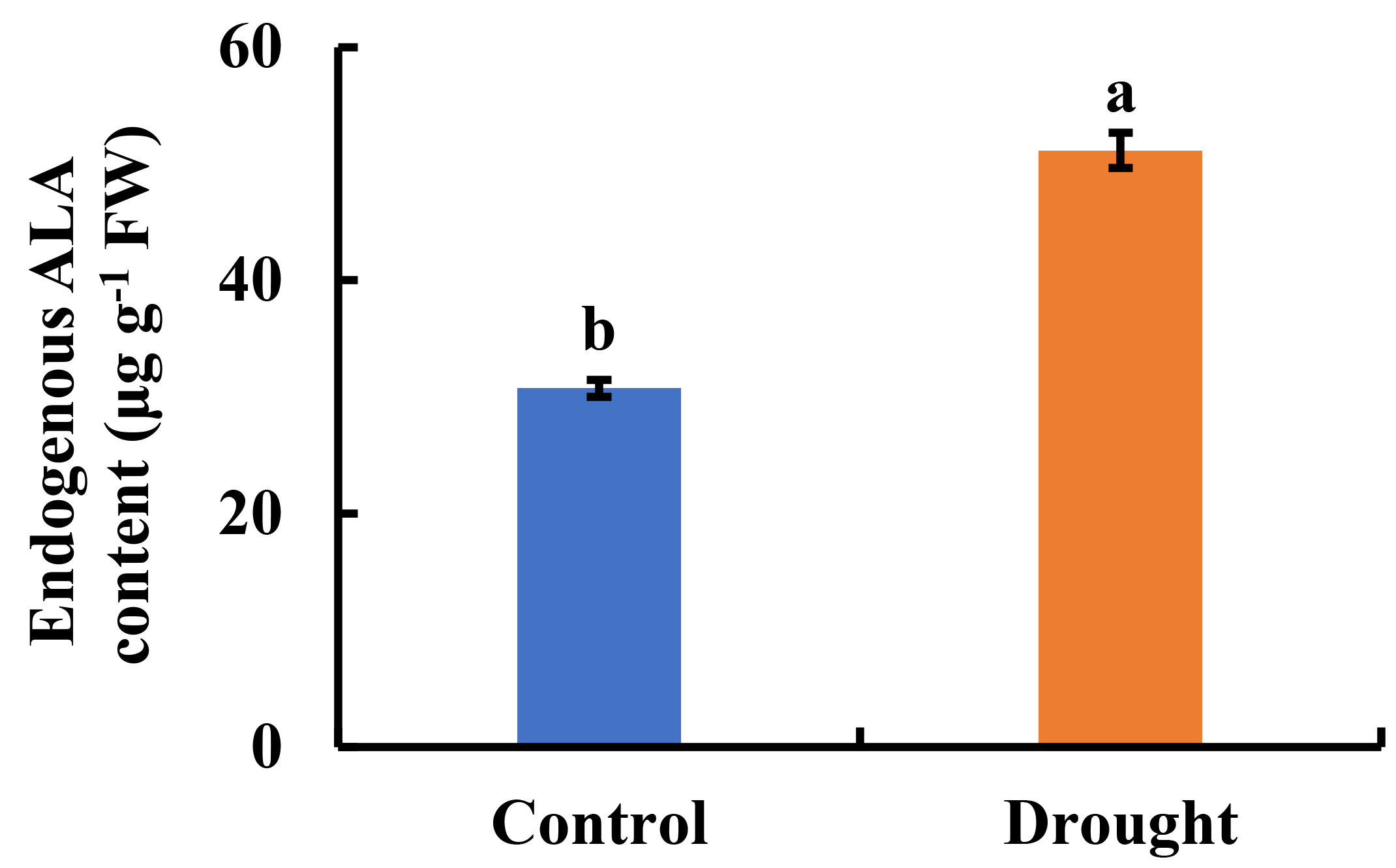


**Fig. S9 Effect of drought stress on the endogenous ALA in epidermis of apple leaves.** The test-tube apple plantlets were cultured on MS medium containing 20% PEG-6000 for 3 days in a growth chamber with temperature of 20-25℃, photoperiod of 16 h a day and PPFD of 240 μmol m^-2^ s^-1^. The data present the means of three independent replicates.
