# Supplementary material for: ALA reverses ABA-induced stomatal closure by modulating PP2AC and SnRK2.6 activity in apple leaves
Source: Hortic Res. 2023 Apr 10;10(6):uhad067. doi: 10.1093/hr/uhad067 (PMC10243991; doi:10.1093/hr/uhad067)
Supplement: Web_Material_uhad067 [file web_material_uhad067.zip › Table S2 Detailed information on apple PP2As.docx]

**Table S2. Detailed information on subunits of apple PP2A holoenzymes**

| Gene subfamily | Gene name | Gene identification | Size (AA) | Genomic position | Theoretical pI | MW (KDa) | Predicted subcellular location |
| --- | --- | --- | --- | --- | --- | --- | --- |
| PP2AA | *MdPP2AA* | 103402718 | 587 | Chr16：3163989-3169431 | 4.80 | 65.54 | nucleus/cytoplasm |
|  | *MdPP2AAβ* | 103414247 | 587 | Chr13：3089263- 3094627 | 4.76 | 65.63 | endoplasmic reticulum |
|  | *MdPP2AAβ-1* | 103437989 | 587 | Chr06：34819546-34824063 | 4.66 | 65.43 | cytoplasm |
|  | *MdPP2AAβ-2* | 103448692 | 124 | Chr 05：39536665-39537830 | 5.50 | 13.45 | peroxisome |
| PP2AB | *MdPP2ABβ-1* | 103453547 | 517 | Chr 09：17739783- 17747149 | 6.04 | 57.68 | cytoskeleton |
|  | *MdPP2ABβ-2* | 103414783 | 517 | Chr 17：19981541- 19990391 | 6.04 | 57.53 | cytoskeleton |
|  | *MdPP2ABβ-3* | 103455205 | 516 | Chr 14：27112005- 27117148 | 6.58 | 58.58 | nucleus |
|  | *MdPP2ABβ-4* | 103455837 | 512 | Chr 15：4122426- 4128415 | 6.17 | 57.31 | nucleus |
|  | *MdPP2ABβ-5* | 103410600 | 461 | Chr 05：24829373- 24831884 | 7.75 | 53.53 | mitochondria |
|  | *MdPP2ABβ-6* | 103440623 | 517 | Chr 08：3340512- 3346709 | 6.33 | 57.64 | cytoskeleton |
|  | *MdPP2ABβ-7* | 103420991 | 450 | Chr 08：3347753- 3355310 | 6.33 | 50.56 | cytoskeleton |
|  | *MdPP2ABβ-8* | 103440602 | 507 | Chr 08：3377285- 3385495 | 6.20 | 57.18 | nucleus |
|  | *MdPP2AB'β* | 103449992 | 497 | Chr 12：17230046-17234393 | 6.68 | 56.86 | cytoplasm |
|  | *MdPP2AB'β-1* | 103427890 | 496 | Chr04：16028283- 16032735 | 6.98 | 57.08 | cytoplasm/mitochondria |
|  | *MdPP2AB'β-2* | 103432588 | 455 | Chr 01：21438183- 21441794 | 6.33 | 52.96 | mitochondria |
|  | *MdPP2AB'γ* | 103442630 | 539 | Chr09：8542674-8546036 | 7.82 | 61.07 | mitochondria |
|  | *MdPP2AB'γ-1* | 103426915 | 541 | Chr17：8792385- 8795975 | 8.46 | 61.28 | mitochondria |
|  | *MdPP2AB'ε* | 103422129 | 210 | Chr 10：20897471- 20898483 | 8.57 | 24.66 | mitochondria |
|  | *MdPP2AB'θ* | 103442169 | 515 | Chr09：1089627-1093303 | 8.22 | 58.83 | chloroplast/nucleus |
|  | *MdPP2AB'θ-1* | 103452092 | 519 | Chr 13：8033521-8045521 | 8.82 | 59.58 | mitochondria |
|  | *MdPP2AB'θ-2* | 114822542 | 514 | Chr 17：1429967-1433783 | 8.54 | 58.44 | nucleus |
|  | *MdPP2AB'θ-3* | 103403117 | 522 | Chr16：7758034- 7761515 | 7.18 | 59.72 | mitochondria |
|  | *MdPP2AB'κ* | 103446316 | 512 | Chr 08：13152933-13155821 | 8.85 | 58.04 | nucleus |
|  | *MdPP2AB'κ-1* | 103456147 | 512 | Chr 12: 17230046-17234393 | 8.60 | 57.95 | nucleus |
|  | *MdPP2AB'κ-2* | 103400479 | 502 | Chr 02: 4972260- 4975589 | 7.97 | 57.12 | mitochondria/chloroplast- mitochondria |
|  | *MdPP2AB'κ-3* | 103426623 | 502 | Chr 02：3476451- 3479935 | 7.95 | 57.24 | nucleus/mitochondria |
|  | *MdPP2AB''TON2* | 103403777 | 476 | Chr 16：14618299- 14622932 | 4.64 | 54.57 | nucleus |
|  | *MdPP2AB''TON3* | 103414625 | 476 | Chr 13：14028664-14034422 | 4.61 | 54.60 | nucleus |
|  | *MdPP2AB''α-1* | 103435725 | 540 | Chr 05：32276314-32282287 | 4.57 | 61.95 | nucleus |
|  | *MdPP2AB''α-2* | 103445606 | 540 | Chr 10：27437405-27443610 | 4.63 | 61.87 | nucleus |
|  | *MdPP2AB''β* | 103434343 | 539 | Chr 04：31738190- 31744257 | 4.65 | 62.10 | nucleus |
|  | *MdPP2AB''δ* | 103419513 | 115 | Chr 12：31906406- 31932025 | 4.35 | 12.29 | chloroplast |
| PP2AC | *MdPP2AC* | 103451899 | 306 | Chr 13：5874613- 5877703 | 4.62 | 35.05 | cytoplasm |
|  | *MdPP2AC-2* | 103443837 | 174 | Chr 06：29414866- 29416856 | 7.30 | 19.69 | extracellular |
|  | *MdPP2AC-3* | 103447007 | 311 | Chr 11：796639- 802022 | 5.08 | 35.62 | cytoplasm |
|  | *MdPP2AC-4* | 103449655 | 313 | Chr 12：5929051- 5935471 | 4.96 | 35.76 | cytoplasm |
|  | *MdPP2AC-5* | 103402956 | 306 | Chr 16：5798139- 5801092 | 4.63 | 35.05 | cytoplasm |
|  | *MdPP2AC-6* | 103415097 | 311 | Chr 03：582469- 587436 | 5.08 | 35.62 | cytoplasm |
|  | *MdPP2AC-7* | 103415254 | 306 | Chr 14：28162644- 28165800 | 4.68 | 35.02 | cytoplasm |
|  | *MdPP2AC-8* | 103422046 | 135 | Mitochondrion：13651- 19217 | 7.37 | 15.19 | chloroplast |
|  | *MdPP2AC-9* | 103434391 | 313 | Chr 14：4896636- 4902261 | 4.96 | 35.77 | cytoplasm |
|  | *MdPP2AC-10* | 103437777 | 306 | Chr 06：32307277- 32310438 | 4.75 | 35.01 | cytoplasm |

Note: MW, Molecular weight; Chr, Chromosome.
