# Supplementary material for: ALA reverses ABA-induced stomatal closure by modulating PP2AC and SnRK2.6 activity in apple leaves
Source: Hortic Res. 2023 Apr 10;10(6):uhad067. doi: 10.1093/hr/uhad067 (PMC10243991; doi:10.1093/hr/uhad067)
Supplement: Web_Material_uhad067 [file web_material_uhad067.zip › Table S3 Research on ALA application in agricultural production since 1984.docx]

**Table S3:** **Research on ALA application in agricultural production since 1984**

| **Year** | **Species** | **Authors** | **Main findings** |
| --- | --- | --- | --- |
| 1984 | Plants | Rebeiz CA, et al. | Photodynamic herbicides at high concentrations |
| 1988 | Insects | Rebeiz CA, et al. | Porphyric insecticides at high concentrations |
| 1993 | Cucumber | Tanaka Y, et al. | Not only a chlorophyll biosynthetic precursor but also involved in biosynthetic regulation |
| 1995 | Rice | Hotta Y, et al. | Improving cold tolerance |
|  | *Spirulina platensis* | Sasaki K, et al. | Promoting growth and photosynthesis |
| 1997 | Tobacco | Zavgorodnyaya, A | Establishing ALAS transgenic plant, which can grow under low light |
|  | Crops | Hotta Y, et al. | Promoting yield, e.g., in rice, radish, garlic, potato, *Vicia faba* |
| 1998 | *Vigna unguiculata* | Bindu RC, Vivekanandan M | Inducing both adventitious buds and adventitious roots, showing both auxin and cytokinin activities |
| 2000 | Cotton | Watanabe, et al. | Promoting salt tolerance |
| 2003 | Spinach | Hotta Y, et al. | ALA-improved salt tolerance dependent on antioxidant activity |
| 2004 | Melon | Wang LJ, et al. | Promotes photosynthesis under low light and chilling conditions with larger stomatal aperture |
|  | Rice | Jung S, et al. | Establishing ALAS transgenic rice; it could not grow under natural light conditions |
|  | Apple | Wang LJ, et al. | Promoting anthocyanin accumulation and fruit coloration |
| 2005 | Pakchoi | Wang LJ, et al. | Promoting seed germination and respiration under salt stress |
|  | Wheat | Lukšienė Z, et al. | Fungal decontamination |
| 2006 | Wheat | Al-Thabet SS | Promoting plant drought tolerance |
|  | Barley | Al-Khateeb SA | Promoting plant drought tolerance |
| 2007 | Soybean | Noriega GO, et al. | Promoting cadmium tolerance |
| 2008 | Oilseed rape | Zhang WF, et al. | Promoting herbicide tolerance |
|  | Tomato | Wang T, et al. | Extending fruit storage after harvest |
| 2009 | Kelp | Tabuchi K, et al. | Promotion of callus propagation |
| 2010 | Arabidopsis | Zhang ZP, et al. | ALA-overproducing transgenic plant with stronger salt tolerance |
|  | Arabidopsis | Maruyama-Nakashita A | Increasing sulfur uptake, transport, and assimilation |
| 2011 | *Rosa hybrida* | Ren ZX, et al. | Promoting flower bud differentiation and flower number |
|  | Pear | Shen M, et al. | As flower thinning agent to thin surplus flowers of fruit trees |
| 2012 | Pakchoi | Wei ZY, et al. | Promoting nitrate absorption, transport, and transfer |
| 2013 | Plants | Akram NA, Ashraf M | Reviewing the biofunctions of ALA in plant stress tolerance as a potential plant growth regulator |
| 2014 | Oilseed rape | Ali B, et al. | Promoting lead tolerance |
|  | Wheat | Bai WB, et al. | Promoting carbohydrate, N, and P transport |
|  | Apple | Chen LH, et al. | Reversing stomatal closure induced by ABA, dark, H_2_O_2_, or Ca^2+^ |
| 2015 | *Elymus nutans* | Fu JJ, et al. | Nitric oxide is involved in mediating ALA-induced chilling tolerance |
|  | Fig | Ma N, et al. | Improving heat tolerance of fig plants |
|  | Oilseed rape | Gill RA, et al. | Promoting chromium tolerance |
|  | Rice | Kanto U, et al. | Enhancing aged seed germination |
| 2016 | Apple | Feng XX, et al. | MADS1 is involved in ALA promotion of anthocyanin biosynthesis |
|  | Fig | An YY, et al. | Improving waterlogging tolerance of fig plants |
|  | Rice | Nguyen H, et al. | Exogenous ALA increased ALA-synthesizing capacity, promoting plant growth, but independent of tetrapyrrole routes |
|  | Apple | Liu LB, et al. | ALA-induced flavonols mediate stomatal opening |
| 2017 | *Beta vulgaris* | Liu LY, et al. | Mitigating sodic-alkaline stress |
|  | Lettuce | Aksakal O, et al. | Alleviating the detrimental effects of UV-B stress |
|  | Rice | Sheteiwy M, et al. | Priming seeds to improve chilling tolerance, like polyamines |
|  | Kidney bean | Taspinar MS, et al. | As an anti-genotoxic agent to reduce genetic damage |
| 2018 | Apple | Xiong LJ, et al. | PP1/PP2A protein phosphatase and microtubule polymerization are involved in ALA-induced stomatal opening |
|  | Cucumber | Wu Y, et al. | Increased salt tolerance is associated with tetrapyrrole routes |
|  | Oilseed rape | Xu L, et al. | Alleviating herbicide-induced damage |
|  |  | Xiong JL, et al. | Improvement of salt tolerance is associated with proline metabolism |
|  |  | Kram NA, et al. | Improvement of drought tolerance is associated with glycine betaine |
|  | Date palm | Alikhani‑Koupaei M, et al. | Moderating bunch wilting induced by high temperature and low humidity |
|  | *Pimpinella anisum* | Tavallali V, Zareiyan F | Influencing essential oil components |
|  | *Matricaria recutita* | Liu X, et al. | Promoting α-bisabolol increase in herbal medicine |
|  | Wheat | Wang YX, et al. | Enhancing transcription of *psbA* and *psbD* of PSII |
|  | Tomato | Liu T, et al. | H_2_O_2_ is involved in ALA-promoted chilling tolerance |
|  | Tomato | Liu T, et al. | NO is involved in H_2_O_2_-mediated ALA-induced chilling tolerance |
| 2019 | Strawberry | Wu WW, et al. | ALA-induced H_2_O_2_ accumulation in roots is responsible for root Na^+^ retention to repress leaf accumulation |
|  |  | An YY, et al. | ALA modulating IAA polar transport to promote root elongation growth |
| 2020 | Strawberry | Cai CY, et al. | Improving drought tolerance by enhancing water conductivity |
|  | *Brassica juncea* | Singh R, et al. | Regulating gene expression involved in Krebs cycle, antioxidant system to confer lead tolerance |
|  | *Zinnia hybrida* | Kai T, et al. | Promoting growth and phytoremediation in oil-contaminated soil |
|  | Maize | Jin X, et al. | Repressing DNA methylation to increase salt tolerance |
| 2021 | Tomato | Wang JW, et al. | Regulating carotenoid metabolism and improving fruit quality |
|  | *Buxus megis-tophylla* | Yang H, et al. | Improving plant growth under urban roadside stress |
| 2022 | Pear | Cao XY, et al. | ABA signaling is involved in ALA-induced anthocyanin  biosynthesis |
|  |  | Sun HL, et al. | Reduced MV-induced damage by induced H_2_O_2_ production |
|  | Apple | Zheng J, et al. | ALA-induced anthocyanin accumulation dependent on expression of genes related to biosynthesis, transport and regulation |
|  |  | Zhang HW, et al. | MdSCL8 negatively regulates ALA-induced flavonol accumulation |
|  |  | Fang X, et al. | ERF78 is involved in ALA-induced anthocyanin accumulation |
|  | Grapevine | Yang YX, et al. | Mitigating copper stress |
|  | Tomato | Zhang ZD, et al. | Alleviating chilling damage by modulating the xanthophyll cycle and nutrient uptake |
|  | Strawberry | He SS, et al. | NO, as well as H_2_O_2_, is involved in ALA-induced Na^+^ retention in roots |
